# Taxonomy, morphology, and phylogeny of a nearly complete nanhsiungchelyid specimen from the Upper Cretaceous of the Nanxiong Basin, China

**DOI:** 10.1186/s13358-025-00385-2

**Published:** 2025-08-05

**Authors:** Yuzheng Ke, Zhongye Shi, Haiyan Tong, Bicheng Li, Yunfei Zhang, Fenglu Han, Walter G. Joyce

**Affiliations:** 1 School of Earth Sciences, China University of Geosciences (Wuhan), Wuhan, China; 2 Department of Geosciences, University of Fribourg, Fribourg, Switzerland; 3 Shanghai Natural History Museum, Shanghai Science and Technology Museum, Shanghai, China; 4 Palaeontological Research and Education Centre, Mahasarakham University, Maha Sarakham, Thailand; 5 Institute of Vertebrate Paleontology and Paleoanthropology, Chinese Academy of Sciences, Beijing, China

**Keywords:** *Nanhsiungchelys*, Plastron, Sexual dimorphism, Phylogeny, Late Cretaceous, Nanxiong Basin

## Abstract

*Nanhsiungchelys*, the type genus of Nanhsiungchelyidae, is an extinct turtle taxon that lived during the Late Cretaceous in southern China. Despite having a relatively abundant fossil record, the plastral morphology of *Nanhsiungchelys* is not well understood. Here, we describe a large specimen of *Nanhsiungchelys* with an associated skull and shell (SNHM 1558) from the Upper Cretaceous of Nanxiong Basin, China. The skull of SNHM 1558 is similar to that of *Nanhsiungchelys yangi*, but the carapace bears a pair of stick-like anterolateral processes. For this reason, we tentatively refer SNHM 1558 to *Nanhsiungchelys* cf. *yangi*. A concave plastron and a narrower posterior lobe indicate that SNHM 1558 may be a male. *Nanhsiungchelys* spp. was previously regarded as the most advanced taxa of Nanhsiungchelyidae, but some morphological characteristics suggest plesiomorphic similarities with the outgroup *Adocus* spp. A phylogenetic analysis using *Adocus* spp. as the outgroup suggests that *Nanhsiungchelys* spp. and *Anomalochelys angulata* form a monophyletic group at the base of Nanhsiungchelyidae. However, this phylogenetic result is not conclusive, as *Nanhsiungchelys* spp. is found deeply nested within the tree when *Sinaspideretes wimani* is employed as the outgroup.

**Supplementary Information:**

The online version contains supplementary material available at 10.1186/s13358-025-00385-2.

## Introduction

Nanhsiungchelyidae Yeh, 1966 is a clade of turtles that lived in Asia and North America during the Cretaceous and one of few turtle groups to go extinct during the Cretaceous-Paleogene extinction event (see Tong (2017)). *Nanhsiungchelys* Yeh, 1966, the type genus of Nanhsiungchelyidae, has so far exclusively been recovered from the Ganzhou and Nanxiong basins of southern China. Although numerous specimens are exhibited in museums that document much variation, only two species, *Nanhsiungchelys wuchingensis* Yeh, 1966 and *Nanhsiungchelys yangi* Ke et al., 2023, have been formally erected (Ke et al., 2023; Tong & Li, 2019; Yeh, 1966). Recently, Tong et al. (2024) and Ke et al. (2024b) described three shells referable to *Nanhsiungchelys* sp., JXGZ(2012)−181, GMNHF10008, and CUGW VH272, from the Upper Cretaceous of Ganzhou Basin. Unfortunately, the lack of associated skull and shell remains hinders a further understanding of their taxonomy.

In the 1970 s, a large nanhsiungchelyid specimen (SNHM 1558) was collected from the Nanxiong Basin, China, which is now housed in the Shanghai Natural History Museum. SNHM 1558 was labelled “*Nanhsiungchelys yehi*” by the staff of the museum, but this name must be considered unvalid, as it has not been formally published. The specimen was first reported by Ye (1994) in his book entitled “*Fossil and recent turtles of China*”. Hirayama et al. (2009) since provided a brief description of this specimen associated with a photograph of its carapace, and Hu et al. (2016) also compared it with other nanhsiungchelyids. Tong (2017) referred this specimen to *Nanhsiungchelys* sp. More recently, Ke et al. (2024b) provided a photograph of the anterior lobe of this specimen showing the possible presence of additional ossifications. No formal systematic description or taxonomic research has otherwise been conducted on SNHM 1558.

In this work, we re-examine SNHM 1558, which contains much valuable information, especially regarding the morphology of the plastron and which provides an opportunity to explore possible sexual dimorphism in *Nanhsiungchelys*. We also reevaluate the most recent phylogenetic matrices of Nanhsiungchelyidae by rescoring all observations.

## Geological setting

The Nanxiong Basin is a faulted basin that spans Guangdong and Jiangxi provinces, China, and contains extensive Mesozoic and Cenozoic strata (Zhang et al., 2013). The Late Cretaceous deposit of the Nanxiong Basin is currently classified as consisting of four formations, namely the Dafeng Formation (Cenomanian to early Campanian), Zhutian Formation (late Campanian), Zhenshui Formation (latest Campanian to early Maastrichtian) and Shanghu Formation (late Maastrichtian) in an ascending order (Guangdong Geological Survey Institute, 2017; Xi et al., 2021). Abundant turtle remains have been collected from the Late Cretaceous beds of the Nanxiong Basin, including *Nanhsiungchelys wuchingensis* and *Nanhsiungchelys yangi* from the Dafeng Formation (Ke et al., 2023; Tong & Li, 2019), *Anosteira lingnanica* Young & Chow, 1962 from the Zhutian Formation and the Pingling Member of the Shanghu Formation (Zhang et al., 2013), and unnamed “large turtles” (without any description or illustration) from the Zhenshui Formation (Zhang et al., 2013).

Specimen SNHM 1558 (field number: 72005) was collected by the Shanghai Natural History Museum in 1972 from south of the town of Nanxiong. Ye (1994) mentioned that the specimen was “from the same horizon” as the holotype of *Nanhsiungchelys wuchingensis* (IVPP V3106). In fact, the holotype of *Nanhsiungchelys wuchingensis* (IVPP V3106) was unearthed from Lashuyuan village within the middle-upper Dafeng Formation (i.e., the “Yuanpu Formation” of Zhang et al. (2013)) (Tong & Li, 2019), while SNHM 1558 was collected from the Zhenshui Formation (Zhang et al., 2013). The Zhenshui Formation is a set of gray-brown and brick-red sandy conglomerates, gravel-bearing sandstones, and sandstones, together with brown–red siltstones and silty mudstones (Guangdong Geological Survey Institute, 2017), and dated from the latest Campanian to the early Maastrichtian (Xi et al., 2021). The coarse sediments associated with SNHM 1558 further support its stratigraphic attribution of the Zhenshui Formation.

## Material and methods

**Fig. 1:**
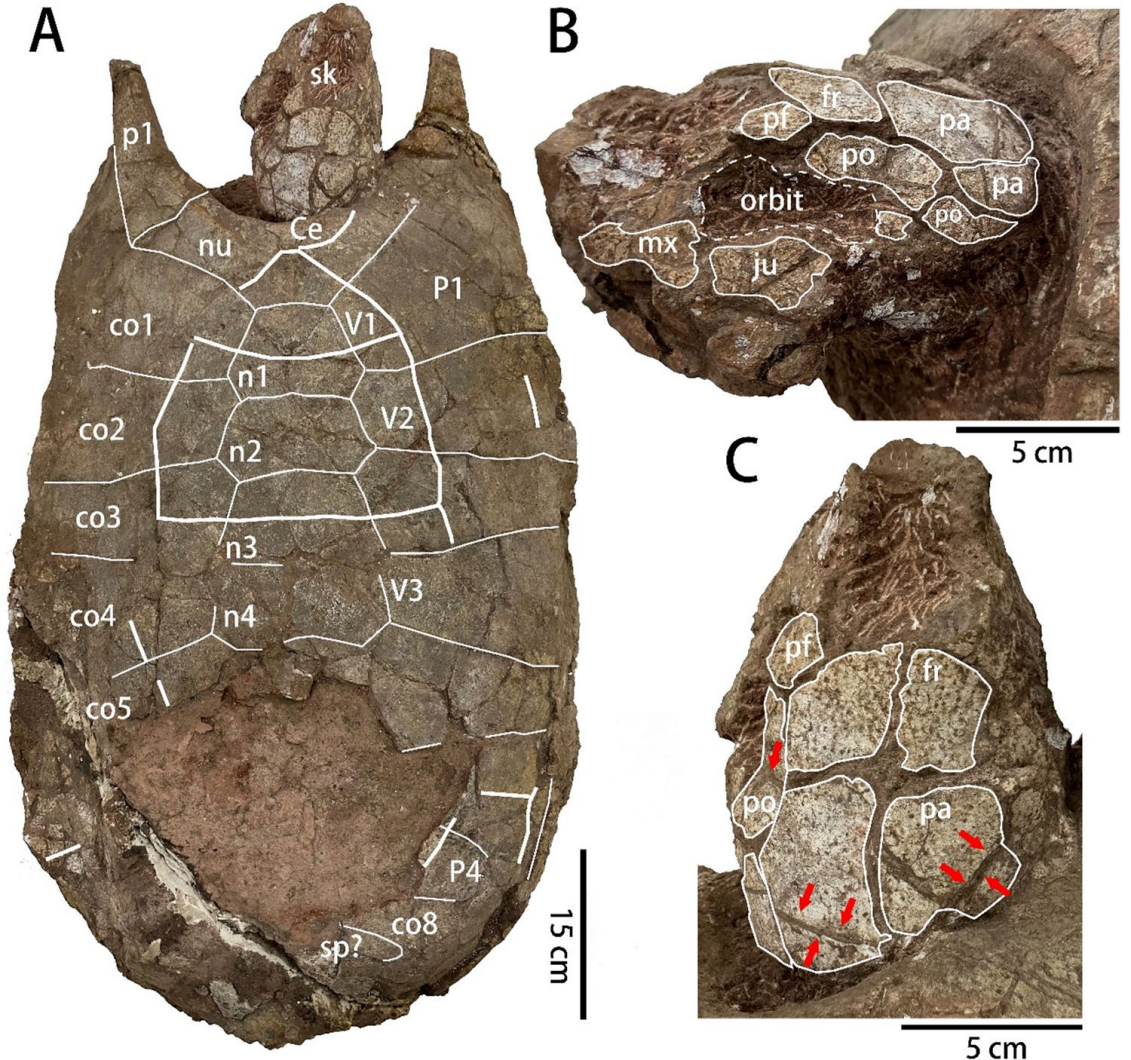
Photograph of the carapace and skull of *Nanhsiungchelys* cf. *yangi* (SNHM 1558). **A** Skull and carapace in dorsal view. **B** Skull in left lateral view. **C** Skull in dorsal view. The thin lines represent sutures, the thick lines represent sulci. Red arrows indicate possible sulci or cracks on the skull. Abbreviations: Ce, cervical scute; co, costal; fr, frontal; ju, jugal; mx, maxilla; n, neural; nu, nuchal; p, peripheral; P, pleural scute; pa, parietal; pf, prefrontal; po, postorbital; sk, skull; sp, suprapygal; V, vertebral scute

**Fig. 2:**
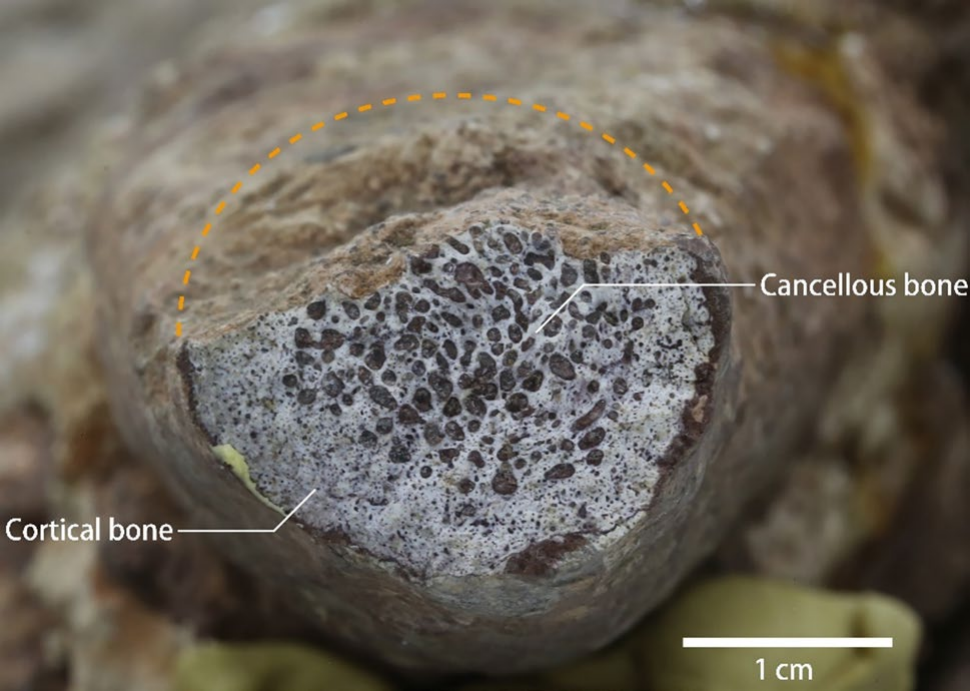
Photograph of the cross section of the right anterolateral process. The dotted line represents the reconstruction of the damaged cross section

**Fig. 3:**
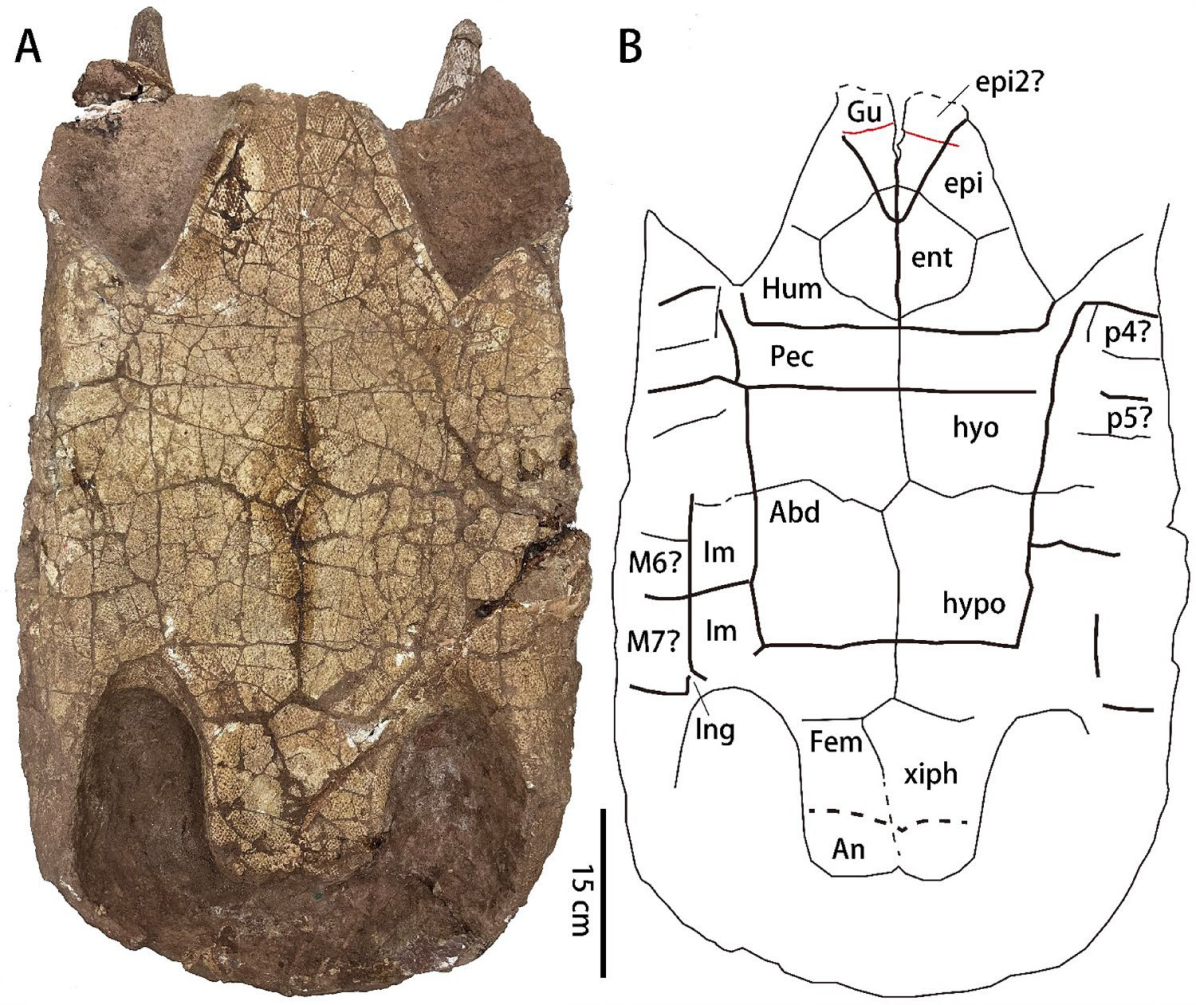
Photograph (**A**) and line drawing (**B**) of the plastron of *Nanhsiungchelys* cf. *yangi* (SNHM 1558) in ventral view. The thin black lines represent sutures, the thin red lines represent possible additional sutures, the thick lines represent sulci, and the dotted lines indicate the reconstruction of poorly preserved areas. Abbreviations: Abd, abdominal scute; An, anal scute; ent, entoplastron; epi, epiplastron; Fem, femoral scute; Gu, gular scute; Hum, humeral scute; hyo, hyoplastron; hypo, hypoplastron; Im, Inframarginal scute; Ing, inguinal scute; M, marginal scute; p, peripheral; Pec, pectoral scute; xiph, xiphiplastron

SNHM 1558 consists of a nearly complete carapace and plastron, as well as the associated skull and lower jaw (Figs. 1, 2 and 3).

The phylogenetic matrix used herein is based on Tong et al. (2024), but was modified as follows. The original state 2 of character 42 (i.e., lateral edges of the first vertebral converge anteriorly) was deleted because it is not related to shape. The character is thus redefined as: first vertebral scute (0) with straight lateral edge; (1) with convex or angled lateral edge. The scoring of *Anomalochelys angulata* Hirayama et al., 2001, *Nanhsiungchelys wuchingensis*, *Nanhsiungchelys* sp. (JXGZ(2012)−181) and *Nanhsiungchelys* sp. (GMNHF10008) is thus changed from 2 to 0 based on a new interpretation of its morphology. Character 53 (i.e., the length of the first costal relative to the second costal) was deleted since length measurements are rarely reported and strongly distorted in images. Two new characters were added. Character 58 evaluates if the nuchal is: (0) narrower than the maximum width of the first vertebral scute; (1) equal to the maximum width of the first vertebral scute; (2) wider than the maximum width of the first vertebral scute. Character 59 evaluates if the pectoro-abdominal sulcus is (0) straight; (1) curved. The full list of characters is provided in Supplementary Information.

Mallon and Brinkman (2018) changed the scorings of *Basilemys gaffneyi* Sullivan et al., 2013 for characters 37 and 38 and of *Basilemys variolosa* (Cope, 1876) for character 32 to polymorphic (i.e., 0/1). As we agree with these observations, we adjusted our current matrix accordingly. We also modified the following scorings. Characters 31 (i.e., presence of extragulars) and 33 (i.e., size and medial contact of extragulars) were changed from 0 to 0/1 and 1 to 0/1, respectively, for *Jiangxichelys neimongolensis* (Brinkman & Peng, 1996), because the extragulars are present in IVPP 020790–5 but absent in 96NMBY-I-14 (Brinkman & Peng, 1996; Brinkman et al., 2015). Characters 18 (i.e., nuchal notch), 23 (i.e., shape of pygal) and 29 (i.e., sulcus between pleural III and marginals VII-IX) were changed from 1 to “?” for “*Zangerlia*” *ukhaachelys* Joyce & Norell, 2005, as most of the carapace is absent (Joyce & Norell, 2005). For *Basilemys sinuosa* (*B. sinuosus*) Riggs, 1906, characters 52 to 57 were scored based on Riggs (1906). Character 28 (i.e., sulcus between pleural I and marginals II and III) was changed for *Hanbogdemys orientalis* (Sukhanov & Narmandakh, 1975) from 1 to 0/1 because the anterior part of this sulcus is located on the peripherals while the posterior part is located on the costo-peripheral suture (Sukhanov, 2000). For *Anomalochelys angulata*, character 17 (i.e., presence of knobby protrusion of the carapace at the position of the first suprapygal) was changed from 0 to “?”, character 29 (i.e., the location of the sulcus between pleural III and marginals VII–IX) was changed from 1 to “?”, and character 39 (i.e., contribution of pectorals to axillary notch) was changed from 1 to “?”, because the relevant parts are missing (Hirayama et al., 2001). For *Kharakhutulia kalandadzei* Sukhanov et al., 2008, character 28 (i.e., sulcus between pleural I and marginals II and III) was changed from 1 to 0 since the sulcus is clearly situated on the peripherals, character 42 (i.e., lateral edge of the first vertebral scute) was changed from 1 to 0 as the first vertebral scute has straight lateral edges, and character 49 was changed from 1 to 0 because an epiplastral beak is absent (Sukhanov et al., 2008). Character 23 (i.e., shape of pygal) was changed for *Jiangxichelys ganzhouensis* Tong & Mo, 2010 from 1 to 0/1 according to the original description of Tong et al. (2016), and character 25 (i.e., contacts of vertebral V with marginals X and XI) was set to 0/1 by reference to NHMG 010415 and JXGZ(2012)−182 (Tong & Mo, 2010; Tong et al., 2016). For “*Zangerlia*” *dzamynchondi* Sukhanov & Narmandakh, 2006, character 38 was changed from 1 to “?” because the sixth marginals are not clear in the illustration, and character 46 was changed from 0 to “?” because the neurals are not visible (Danilov et al., 2013). Character 19 (i.e., shape and size of nuchal) was changed for *Nanhsiungchelys yangi* from 0 to “?” because the nuchal is missing (Ke et al., 2023). Character 46 (i.e., width of the neurals) was changed for *Basilemys morrinensis* Mallon & Brinkman, 2018 from 0 to “?” because neurals cannot be identified (Mallon & Brinkman, 2018). For *Yuchelys nanyangensis* Tong et al., 2012, character 10 was changed from 0 to “?” (i.e., central morphology of the eighth cervical is invisible), character 26 was changed from 1 to 0 (i.e., vertebral V only partially covers suprapygals), character 27 was changed from 1 to 0 (i.e., vertebral V does not reach peripheral X), character 31 was changed from “?” to 0 (i.e., extragular is visible), and character 55 was changed from “?” to 0 (i.e., pleurals 2–4 are not narrow) following Tong et al. (2012). Character 21 (i.e., presence of eight neurals) was changed for *Xianyuechelys yingliangi* Ke et al., 2024a from 1 to “?” since the holotype only includes seven neurals but the first suprapygal is probably fused with the eighth neural, and character 42 (i.e., lateral edge of the first vertebral scute) was changed from 1 to 0 as the first vertebral scute has straight lateral edges (Ke et al., 2024a). For *Nanhsiungchelys* sp. (JXGZ(2012)−181), character 17 (i.e., if knobby protrusion of the carapace occurs at the position of the first suprapygal) was changed from 0 to “?” due to the poor preservation of this specimen (Tong et al., 2024). Character 51 was changed for *Nanhsiungchelys* sp. (GMNHF10008) from “?” to 0 since the posterior part of the first suprapygal is not significantly wider than the anterior part (Tong et al., 2024). Finally, character 35 of *Adocus* spp. was changed from 0 to 1 since the gulars overlap onto entoplastron based on CCM 60-15 (Meylan & Gaffney, 1989). The matrix was expanded through the addition of *Sinaspideretes wimani* Young & Chow, 1953 (Tong et al., 2014).

Two parsimony phylogenetic analyses were performed using the software TNT 1.5 (Goloboff et al., 2016). The first analysis included the 20 nanhsiungchelyids and *Adocus* spp. as the outgroup. The second analysis retained *Adocus* spp., but added *Sinaspideretes wimani* as the outgroup. These analyses were conducted using a traditional search with 1000 replicates. A tree bisection reconnection (TBR) swapping algorithm was employed and 1000 trees were saved per replicate. All characters were treated as unordered and of equal weight. Standard bootstrap support values were calculated using a traditional search with 1000 replicates.

## Systematic palaeontology

Testudines Batsch, 1788

Cryptodira Cope, 1868 (sensu Joyce et al., 2020).

Nanhsiungchelyidae Yeh, 1966 (sensu Joyce et al., 2021).

*Nanhsiungchelys* Yeh, 1966

*Nanhsiungchelys yangi* Ke et al., 2023

*Nanhsiungchelys* cf. *yangi.*

**Referred specimen.** SNHM 1558, a nearly complete carapace and plastron with associated skull and lower jaw.

**Locality and horizon.** South of the town of Nanxiong, Guangdong Province, China; Upper Cretaceous Zhenshui Formation (latest Campanian to early Maastrichtian).

## Description and comparison

The skull is poorly preserved. The anterior part of the skull roof is damaged (Fig. 1A). Only the bones in dorsal and left sides of the skull can be identified with some confidence, but the large spaces between the bones limit the understanding of their shape and size (Fig. 1B, C). The snout is nearly triangular in dorsal view, similar to *Nanhsiungchelys yangi* (Ke et al., 2023), but contrary to *Nanhsiungchelys wuchingensis*, which has an unusual trumpet shape snout (Tong & Li, 2019). Most preserved parts of the maxilla are located anterior to the orbit, as in *Nanhsiungchelys wuchingensis* and *Nanhsiungchelys yangi* (Ke et al., 2023; Tong & Li, 2019). The jugal constitutes the lower rim of the orbit. Due to the poor state of preservation, the shape and size of the cheek emargination cannot be clearly discerned. The postorbital is elongated anteroposteriorly and forms the posterodorsal and posterior rims of the orbit, as in *Nanhsiungchelys wuchingensis* and *Nanhsiungchelys yangi* (Ke et al., 2023; Tong & Li, 2019). The skull roof includes a partial prefrontal, a pair of pentagonal frontals, and a pair of parietals (Fig. 1C). The prefrontal forms part of the dorsal rim of the orbit and contacts the postorbital and frontal posteriorly. Thus, the frontal is entirely excluded from the orbit, similar to other nanhsiungchelyids (Brinkman et al., 2015; Joyce & Norell, 2005; Ke et al., 2023; Tong & Li, 2019). The frontals are smaller than the parietals, as in *Nanhsiungchelys yangi* (Ke et al., 2023), but in contrast to *Nanhsiungchelys wuchingensis* (Tong & Li, 2019). The outline of the temporal emargination cannot be identified with confidence, but likely resembles the shallow condition of *Nanhsiungchelys wuchingensis* and *Nanhsiungchelys yangi* (Ke et al., 2023; Tong & Li, 2019).

The lower jaw is exposed in ventral view. The angle between the left and right branches is ~ 70° in SNHM 1558, wider than in *Nanhsiungchelys wuchingensis* and *Nanhsiungchelys yangi* (< 55°) (Ke et al., 2023; Tong & Li, 2019). The unsatisfactory preservation limits more comparison.

The carapace is 88 cm long and 50 cm wide (maximum width at the level of the sixth costal), with a length to width ratio of ~ 1.76 (Fig. 1A). The nuchal is large, nearly trapezoidal, and signifiantly wider than the first vertebral scute, forming the central part of the deep nuchal emargination. The suture between the nuchal and the first neural is straight, as in *Anomalochelys angulata* and *Nanhsiungchelys* spp. (Hirayama et al., 2001; Tong & Li, 2019; Tong et al., 2024), but differing from the curved condition of *Xianyuechelys yingliangi* (Ke et al., 2024a). The preserved neurals (i.e., the first to fourth neurals) are wider than long, as in *Nanhsiungchelys* spp. (Tong & Li, 2019; Tong et al., 2024). On the contrary, the first to fourth neurals of *Anomalochelys angulata* are elongated or as long as wide (Hirayama et al., 2001), and the other nanhsiungchelyids usually have elongated neurals (e.g., *Kharakhutulia kalandadzei*; Sukhanov et al., 2008). The first to fourth neurals are wide hexagons with short posterolateral sides. The differentiated neural series that occurs in *Nanhsiungchelys* sp. (JXGZ(2012)−181 and GMNHF10008, Tong et al., 2024) is not obvious in SNHM 1558. A total of eight pairs of costals could be identified. The second to the eighth costals are alternating, as in *Nanhsiungchelys* sp. and *Xianyuechelys yingliangi* (Ke et al., 2024a; Tong et al., 2024). A possible suprapygal appears posterior to the eighth costal, but the poor state of preservation limits an accurate identification. The first peripheral is elongated and forms the anterolateral process. These processes are stick-like, as in *Nanhsiungchelys* sp. (JXGZ(2012)−181, Tong et al., 2024). By contrast, the processes of *Nanhsiungchelys wuchingensis*, *Nanhsiungchelys yangi*, and *Nanhsiungchelys* sp. (GMNHF10008) are flat-like (Ke et al., 2023; Tong & Li, 2019; Tong et al., 2024). Although *Anomalochelys angulata* has a pair of similar processes, these elements are only formed by the nuchal (Hirayama et al., 2001). The end of the right process was broken and shows a clear cross section, suggesting the cortical bone and a greater proportion of cancellous bone (Fig. 2).

The cervical scute is crescent-shaped and estimated to be 3.5 times wider than long. This ratio is similar to that of *Nanhsiungchelys* sp. (JXGZ(2012)−181), but wider than that of *Nanhsiungchelys wuchingensis* and *Nanhsiungchelys* sp. (GMNHF10008) (Tong & Li, 2019; Tong et al., 2024). The first vertebral scute is roughly triangular with the lateral edges converging anteriorly, as in *Anomalochelys angulata* and *Nanhsiungchelys* spp. (Hirayama et al., 2001; Tong & Li, 2019; Tong et al., 2024). The second vertebral scute is a wide pentagon. The shape of the third to fifth vertebral scutes cannot be well identified, but these elements are also wider than long. The anterior and right margins of the fourth pleural scute could be identified, which is narrow with the lateral sulcus situates on the costals, as in *Nanhsiungchelys* sp. (JXGZ(2012)−181) (Tong et al., 2024).

The plastron is well preserved with most sutures and sulci visible (Fig. 3). The plastron is significantly concave in the middle. The epiplastra are located at the anterior end of the plastron. Their anterior margin protrudes farther anterior than the nuchal at its emargination. The left and right epiplastra are disarticulated at the midline, which may be caused by taphonomic compression. Two lines horizontally cross the epiplastra that may represent sutures of additional ossifications (red lines in Fig. 3B) (Ke et al., 2024b). The entoplastron is large and diamond-shaped. Its posterior end is located posterior to the axillary notch. Both hyoplastron and hypoplastron are large and make a similar contribution to the bridge. The suture between the hyoplastron and the hypoplastron is sinuous, as in *Nanhsiungchelys wuchingensis* (Tong & Li, 2019). The posterior lobe is narrow and elongate, with a concave posterior rim. The xiphiplastron is nearly trapezoidal, but the anterior margin is irregular. The bridge is long, clearly longer than the anterior or the posterior lobe.

The paired gular scutes are triangular, with the posterior edge extending deeply onto the entoplastron, as in *Nanhsiungchelys* sp. (CUGW VH272, Ke et al., 2024b). In comparison, *Nanhsiungchelys wuchingensis* has a single gular scute (Tong & Li, 2019). The extragular scute is lacking, as in other *Nanhsiungchelys* specimens (Ke et al., 2023, 2024b; Tong & Li, 2019). The humeropectoral sulcus is straight except the lateral ends, and does not intersect onto the entoplastron, which is unique among nanhsiungchelyids. Other nanhsiungchelyids have an anteriorly convex humeropectoral sulcus extending onto the entoplastron (Brinkman & Nicholls, 1993; Brinkman et al., 2015; Danilov et al., 2013; Joyce & Norell, 2005; Mallon & Brinkman, 2018; Sukhanov, 2000; Sukhanov et al., 2008; Tong & Li, 2019; Tong et al., 2012, 2016). The pectoral scute is extremely short, but a small part extends anteriorly to form the rim of the axillary notch, different from that of *Nanhsiungchelys wuchingensis* (Tong & Li, 2019). The abdominal scute is large and square. The femoral scute is trapezoidal. The anal scute is small, wider than long. At least two inframarginal scutes could be identified between the abdominal and marginal scutes, which are wider than those of *Nanhsiungchelys wuchingensis* (Tong & Li, 2019). A small inguinal scute is located near the inguinal notch.

## Discussion

### Taxonomy

SNHM 1558 was referred to Nanhsiungchelyidae by Ye (1994) and Hirayama et al. (2009). A decisive character is that the shell of SNHM 1558 is covered by a network of pits and ridges (Fig. 3A). Although the slender anterolateral processes resemble those of *Anomalochelys angulata* from Japan (Hirayama et al., 2001), Tong (2017) included SNHM 1558 in *Nanhsiungchelys* because these processes are formed by the first peripheral, coupled with the sculpture on the skull and the plastral morphology that are conform to this genus. The shell of SNHM 1558 presents *Nanhsiungchelys* characters, such as the wide neurals and vertebral scutes, the alternating costals, the narrow fourth pleural scute, and the possible additional ossifications on the epiplastra.

*Nanhsiungchelys* contains two valid species, *Nanhsiungchelys wuchingensis* and *Nanhsiungchelys yangi* (Ke et al., 2023, 2024b; Tong & Li, 2019). Three undeterminate specimens (JXGZ(2012)−181, GMNHF10008, and CUGW VH272) have furthermore been referred to this genus as *Nanhsiungchelys* sp. (Ke et al., 2024b; Tong et al., 2024).

SNHM 1558 can be distinguished from *Nanhsiungchelys wuchingensis* in which the snout is trumpet-shaped in dorsal view, the frontal is larger than the parietal, the anterolateral process is flat, the width of the nuchal equals to that of the first vertebral scute, the cervical scute is small and square, single gular scute, the entoplastron is slightly more anteriorly placed, and the humeropectoral sulcus intersects the entoplastron (Tong & Li, 2019).

The overall carapace morphology of SNHM 1558 is comparable with that of *Nanhsiungchelys* sp. (JXGZ(2012)−181 and GMNHF10008). SNHM 1558 seems to be closer to JXGZ(2012)−181 due to the stick-like anterolateral processes, the deep cervical emargination, and the nuchal that is wider than the first vertebral scute (Tong et al., 2024). However, some details are different. The most important feature is that both JXGZ(2012)−181 and GMNHF10008 have obvious neural differentiation (Tong et al., 2024). In addition, GMNHF10008 has a shallower cervical emargination, a pair of flat anterolateral processes, and the width of the nuchal equals to that of the first vertebral scute (Tong et al., 2024). Unfortunately, both JXGZ(2012)−181 and GMNHF10008 lack the skull and the plastron, thus hindering more comparison with SNHM 1558.

*Nanhsiungchelys* sp. (CUGW VH272) only includes a fragmentary anterior lobe (Ke et al., 2024b). Both CUGW VH272 and SNHM 1558 have an entoplastron more posteriorly placed relative to *Nanhsiungchelys wuchingensis*, with a longer epiplastral midline length. More comparison is limited due to the poor preservation of CUGW VH272.

The remaining species within this genus is *Nanhsiungchelys yangi*, which is based on an incomplete specimen with a skull and anterior end of the shell from the Nanxiong Basin (Ke et al., 2023, 2024b). *Nanhsiungchelys yangi* and SNHM 1558 are similar in their overall skull morphology, especially the triangular snout in dorsal view, the smaller frontal, the larger parietal, as well as the relative locations of the maxilla, the jugal and the prefrontal. However, *Nanhsiungchelys yangi* has a narrower lower jaw (with a smaller angle between the left and right rami) and a pair of flat anterolateral processes (Ke et al., 2023). Therefore, we assign SNHM 1558 as *Nanhsiungchelys* cf. *yangi*.

### Morphology and the possible sexual dimorphism

Some nanhsiungchelyids have distinct scute sulci on the skull roof, such as *Jiangxichelys neimongolensis* and *Nanhsiungchelys wuchingensis* (Brinkman et al., 2015; Tong & Li, 2019). In the holotype of *Nanhsiungchelys wuchingensis* (IVPP V3106), the sulci cross the prefrontal, the frontal, the parietal, the maxilla, the jugal, and the quadratojugal (Tong & Li, 2019). In contrast, no cranial scute sulcus can be observed in *Nanhsiungchelys yangi* (Ke et al., 2023), possibly representing a diagnostic difference with *Nanhsiungchelys wuchingensis*. As for the skull of SNHM 1558, although there are several deep grooves on the postorbital and the parietal (arrows in Fig. 1C), it is hard to determine if they are sulci or cracks.

The discovery of SNHM 1558 also provides a chance to explore the plastral morphology of *Nanhsiungchelys*. The lack of extragular scutes of *Nanhsiungchelys* contrasts with most other nanhsiungchelyid turtles, such as *Basilemys praeclara* Hay, 1910, *Hanbogdemys orientalis*, *Kharakhutulia kalandadzei*, *Yuchelys nanyangensis*, and “*Zangerlia*” *dzamynchondi* (Brinkman & Nicholls, 1993; Danilov et al., 2013; Sukhanov, 2000; Sukhanov et al., 2008; Tong et al., 2012). The anterior edge of the pectoral scute is straight in SNHM 1558, whereas the humeropectoral sulcus is convex to intersect the entoplastron in other nanhsiungchelyids (Brinkman & Nicholls, 1993; Brinkman et al., 2015; Danilov et al., 2013; Mallon & Brinkman, 2018; Sukhanov, 2000; Sukhanov et al., 2008; Tong et al., 2012, 2016). SNHM 1558 provides some evidence for the presence of additional ossifications of the epiplastra in *Nanhsiungchelys*, a hypothesis put forward by Ke et al. (2024b), but we cannot exclude the possibility for the moment that the transverse lines we observe across the anterior lobe are the result of taphonomic crushing below the epiplastral lip.

The plastron of SNHM 1558 is concave, suggesting this individual is a male. In living turtles, especially the terrestrial species, males have a concave plastron to facilitate mating, whereas the plastron of females is flat (Pritchard, 2007). The narrow posterior lobe of SNHM 1558 may also be a sexual character of the male. In *Jiangxichelys ganzhouensis*, the shape of the posterior lobe is variable and that has been suggested as a result of sexual dimorphism (Tong et al., 2016). The holotype (NHMG 010415), with a narrow posterior lobe and concave plastron, is thus probably a male, while other *Jiangxichelys ganzhouensis* specimens with flat plastron and wider posterior lobe are likely females (Tong & Mo, 2010; Tong et al., 2016).

An important feature of *Nanhsiungchelys* spp. is the pair of anterolateral processes of the carapace formed by the first peripherals, which are present in two morphotypes (i.e., stick-like or flat-like). Associated with the other sexual characters (e.g., a concave plastron and narrow posterior lobe), the stick-like anterolateral processes in SNHM 1558 are possibly also a male character, whereas the flat processes may belong to females. Similar anterolateral processes are present on the carapace of *Stupendemys geographicus* Wood, 1976 from the Miocene of South America. Cadena et al. (2020) suggested that the specimens with the processes are males and those without processes are females, and such “horns” serve as weapons in the combat behavior of males. In *Nanhsiungchelys*, the stick-like processes, more solid than the flat processes, probably have the same function. More complete specimens with associated carapace and plastron are needed to further explore this hypothesis.

### Phylogeny

**Fig. 4:**
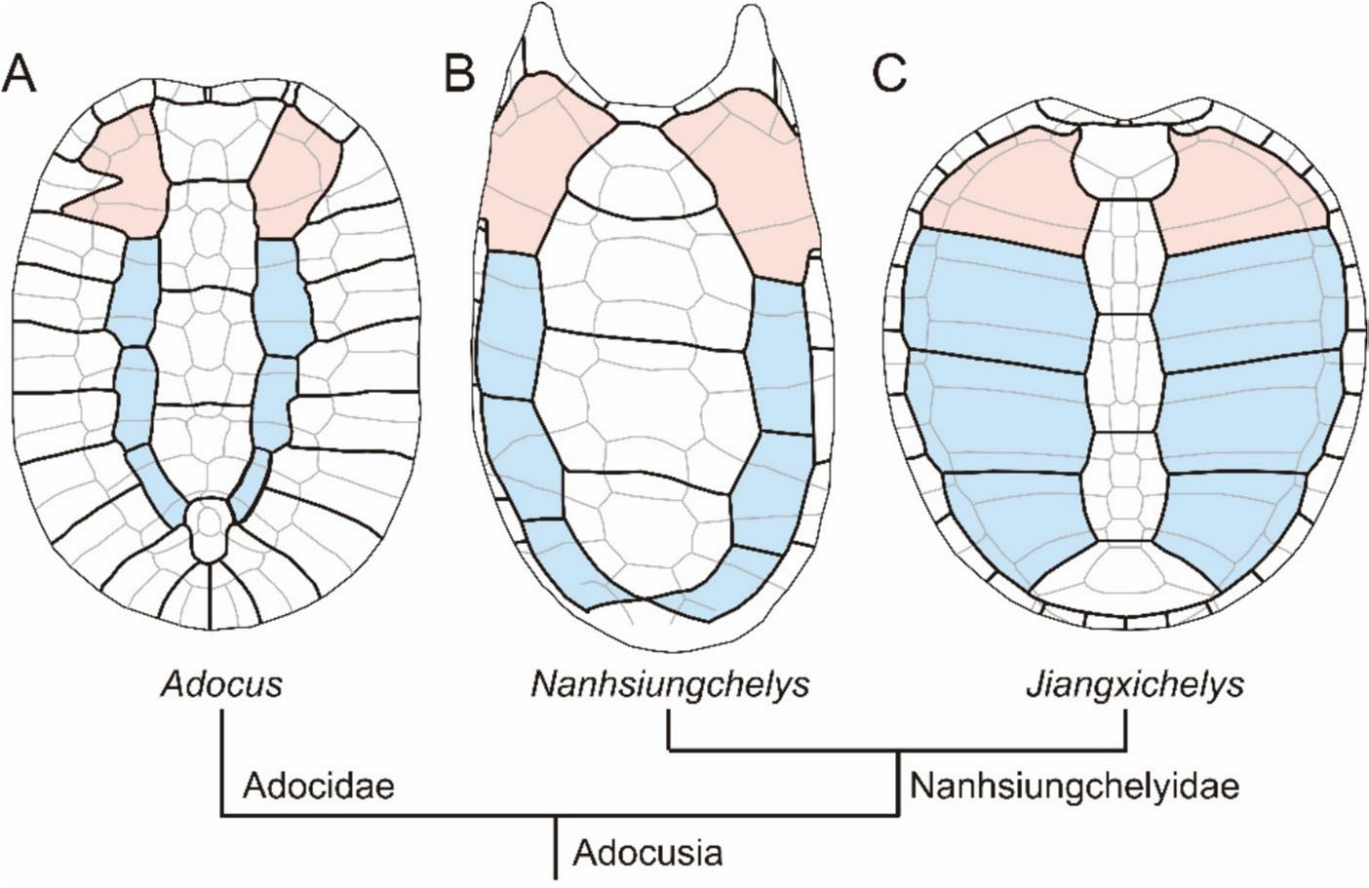
Carapace reconstruction of *Adocus amtgai* (**A**, after Syromyatnikova et al. (2013)), *Nanhsiungchelys* sp. (**B**, after Tong et al. (2024)), and *Jiangxichelys ganzhouensis* (**C**, after Tong et al. (2016)) shown on the simplified phylogenetic tree. Black lines represent the sulci between scutes, grey lines represent the sutures, pink surfaces represent the first pleural scute, and blue surfaces represent the second to the fourth pleural scutes

The phylogeny of Nanhsiungchelyidae has been investigated by many researchers over the course of the last three decades (e.g., Brinkman et al., 2015; Danilov et al., 2013; Hirayama et al., 2001; Joyce & Norell, 2005; Ke et al., 2023, 2024a; Sukhanov et al., 2008; Tong & Li, 2019; Tong et al., 2024). The resulting analyses typically conclude that *Zangerlia testudinimorpha* Mlynarski, 1972 or *Kharakhutulia kalandadzei* are the most basal nanhsiungchelyids, whereas *Nanhsiungchelys* spp. and *Anomalochelys angulata* are regarded as the most advanced. However, some morphological characteristics of *Nanhsiungchelys* suggest plesiomorphic similarities with the outgroup, *Adocus* spp. Particularly, Tong et al. (2024) reported that *Nanhsiungchelys* sp. has narrow second to fourth pleurals and higher posterior marginals, which are also present in *Adocus amtgai* Narmandakh, 1985 (Syromyatnikova et al., 2013), but not in other nanhsiungchelyids (Fig. 4). Second, *Nanhsiungchelys* cf. *yangi* (SNHM 1558; this paper) has an extremely narrow pectoral, similar to that of *Adocus* spp. (Meylan & Gaffney, 1989; Syromyatnikova et al., 2013).

**Fig. 5:**
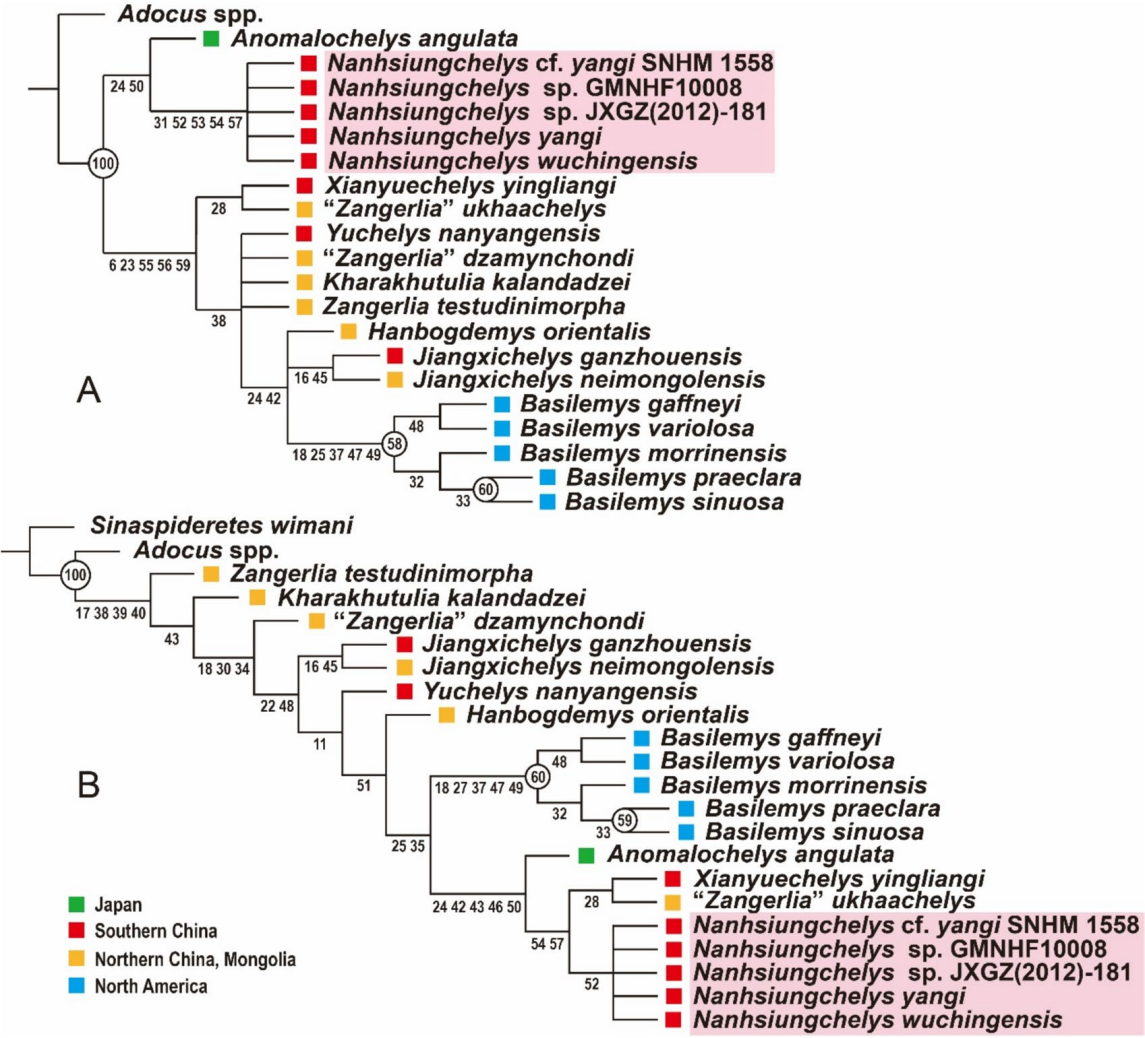
Phylogenetic hypotheses of Nanhsiungchelyidae. **A**, strict consensus tree including 20 nanhsiungchelyids and *Adocus* spp. **B**, strict consensus tree including 20 nanhsiungchelyids, *Adocus* spp., and *Sinaspideretes wimani*. Numbers in circles are bootstrap support values (the values less than 50 are not shown here), and numbers below nodes are character numbers of common synapomorphies

Our first phylogenetic analysis retrieved 12 most parsimonious trees with a length of 97 steps, a consistency index (CI) of 0.649, and a retention index (RI) of 0.719. The five species/specimens of *Nanhsiungchelys* form a monophyletic group (Fig. 5A), supported by character 31 (i.e., absence of extragulars), character 52 (i.e., the first peripheral enlarged to significantly form the anterolateral process), character 53 (i.e., presence of differentiated neural series), character 54 (i.e., alternating costals), and character 57 (i.e., posterior marginals clearly higher than corresponding peripherals). However, the result does not support an exclusive sister group relationship between *Nanhsiungchelys* cf. *yangi* and *Nanhsiungchelys yangi*, compared to the other species of *Nanhsiungchelys*. *Nanhsiungchelys* spp. and *Anomalochelys angulata* form a clade supported by character 24 (i.e., anterior side of the first vertebral only in contact with the cervical) and character 50 (i.e., length to width ratio of the carapace ≥ 1.6). This phylogeny finally suggests that the *Nanhsiungchelys*-*Anomalochelys* clade is the sister to all other nanhsiungchelyid taxa. As *Anomalochelys angulata* and *Kharakhutulia kalandadzei* have been reported to originate from Cenomanian deposits (Hirayama et al., 2001; Sukhanov et al., 2008), this implies that two distinct nanhsiungchelyid lineages were present at least since the Cenomanian (100.5 ~ 93.9 Ma).

Unlike the former results of Ke et al. (2024a) and Tong et al. (2024), *Xianyuechelys yingliangi* is distantly related with *Nanhsiungchelys* spp. in the present tree, instead forming a clade with “*Zangerlia*” *ukhaachelys* (Fig. 5A). This is supported by character 28 (i.e., sulcus between pleural I and marginals II and III not situated on peripherals). “*Zangerlia*” *ukhaachelys* was established based on a juvenile specimen from the Campanian of Ukhaa Tolgod, Mongolia (Joyce & Norell, 2005) and is therefore close in time and space with *Zangerlia testudinimorpha* (Campanian, Nemegt Basin) (Danilov et al., 2013; Mlynarski, 1972). The shell morphology of the above two species is highly similar. Although Joyce and Norell (2005) mentioned “*Zangerlia*” *ukhaachelys* differing from *Zangerlia testudinimorpha* by a complete row of four sub-equally sized inframarginals, it should be noted that intraspecific variation in inframarginal scute count is common among turtles (e.g., the number of inframarginals is polymorphic in *Basilemys gaffneyi*, see the comment of Mallon and Brinkman (2018)). Therefore, the possibility that “*Zangerlia*” *ukhaachelys* is a juvenile of *Zangerlia testudinimorpha* cannot be excluded, but this synonymy is beyond the scope of this article.

Our second phylogenetic analysis suggests that the phylogenetic results discussed above are perhaps not conclusive. This analysis used the Late Jurassic taxon *Sinaspideretes wimani*, a plausible early adocusian (Tong et al., 2014) as the outgroup. This analysis retrieved three most parsimonious trees with a length of 99 steps, a CI of 0.636 and a RI of 0.723. The result retains *Adocus* spp. in a basal position, but *Nanhsiungchelys* spp. is once again found deeply nested within the tree (Fig. 5B), somewhat similar to the result of Mallon and Brinkman (2018). If this result is correct, the narrow pleurals 2–4 and the high posterior marginals present in *Adocus* spp. and *Nanhsiungchelys* spp. were acquired independently. This result at the very least suggests that ingroup relationships strongly depend on the selection of outgroups. We trust that additional specimens and characters will help further address this issue in the future.

## Conclusions

A large nanhsiungchelyid specimen (SNHM 1558) from the Upper Cretaceous Zhenshui Formation of Nanxiong Basin is described here and referred to *Nanhsiungchelys* cf. *yangi*. It has a skull similar to that of *Nanhsiungchelys yangi*, in that the snout is triangular in dorsal view, the frontal smaller, and the parietal larger. With a concave plastron and narrow posterior lobe, SNHM 1558 is likely a male. The stick-like anterolateral processes of SNHM 1558, different from the flat processes of *Nanhsiungchelys yangi*, maybe a sexual dimorphism character. Finally, our phylogenetic result supports the monophyly of *Nanhsiungchelys* spp. and *Anomalochelys angulata* as sister to all remaining nanhsiungchelyids. However, this phylogenetic result is not conclusive as *Nanhsiungchelys* spp. is found deeply nested when *Sinaspideretes wimani* is used as the outgroup.

## Supplementary Information

Supplementary material 1: Phylogenetic matrix

Supplementary material 2: Original photos of SNHM 1558

## Data Availability

Data is provided within the supplementary information files.

## Abbreviations

CCM: Carter County Museum, Ekalaka, USA
CUGW: China University of Geosciences (Wuhan), Wuhan, China
GMNHF: Ganzhou Museum, Ganzhou, China
IVPP: Institute of Vertebrate Paleontology and Paleoanthropology, Chinese Academy of Sciences, Beijing, China
JXGZ: Mineral Resources Administration of Ganzhou, Ganzhou, China
NHMG: Natural History Museum of Guangxi Zhuang Autonomous Region, Nanning, China
NMB: Nei Menggu Bowuguan (= Inner Mongolia Museum), Hohhot, China
SNHM: Shanghai Natural History Museum, Shanghai, China
