# Supplementary figures and images for: Taxonomy, morphology, and phylogeny of a nearly complete nanhsiungchelyid specimen from the Upper Cretaceous of the Nanxiong Basin, China
Source: Swiss J Palaeontol. 2025 Aug 5;144(1):48. doi: 10.1186/s13358-025-00385-2 (PMC12325503; doi:10.1186/s13358-025-00385-2)

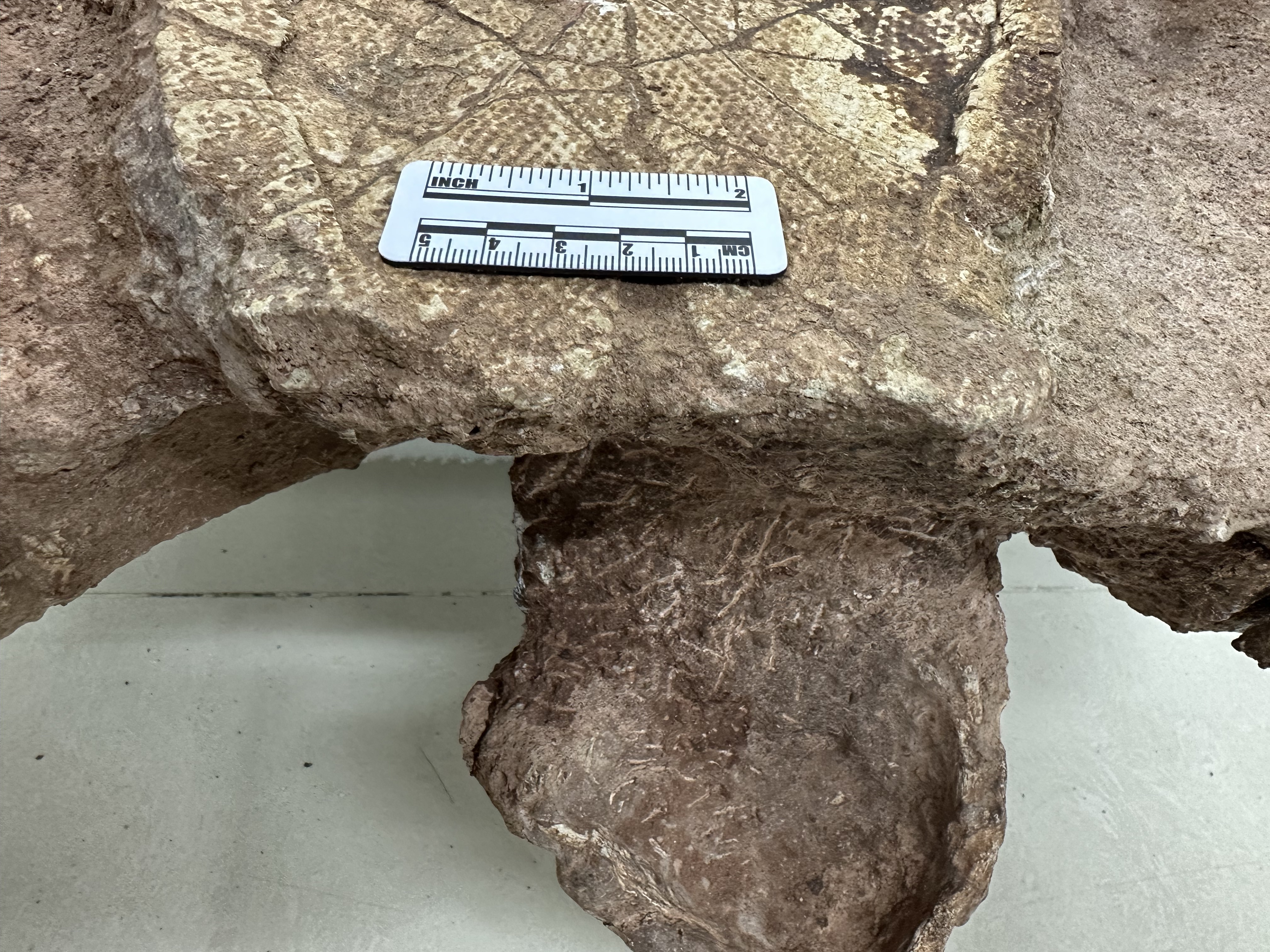

Supplement: Supplementary file 2 — Supplementary material 2: Original photos of SNHM 1558 [file 13358_2025_385_MOESM2_ESM.zip › original photos of SNHM1558/e53477192a109cfe3df8a19edd7d2186.jpg]

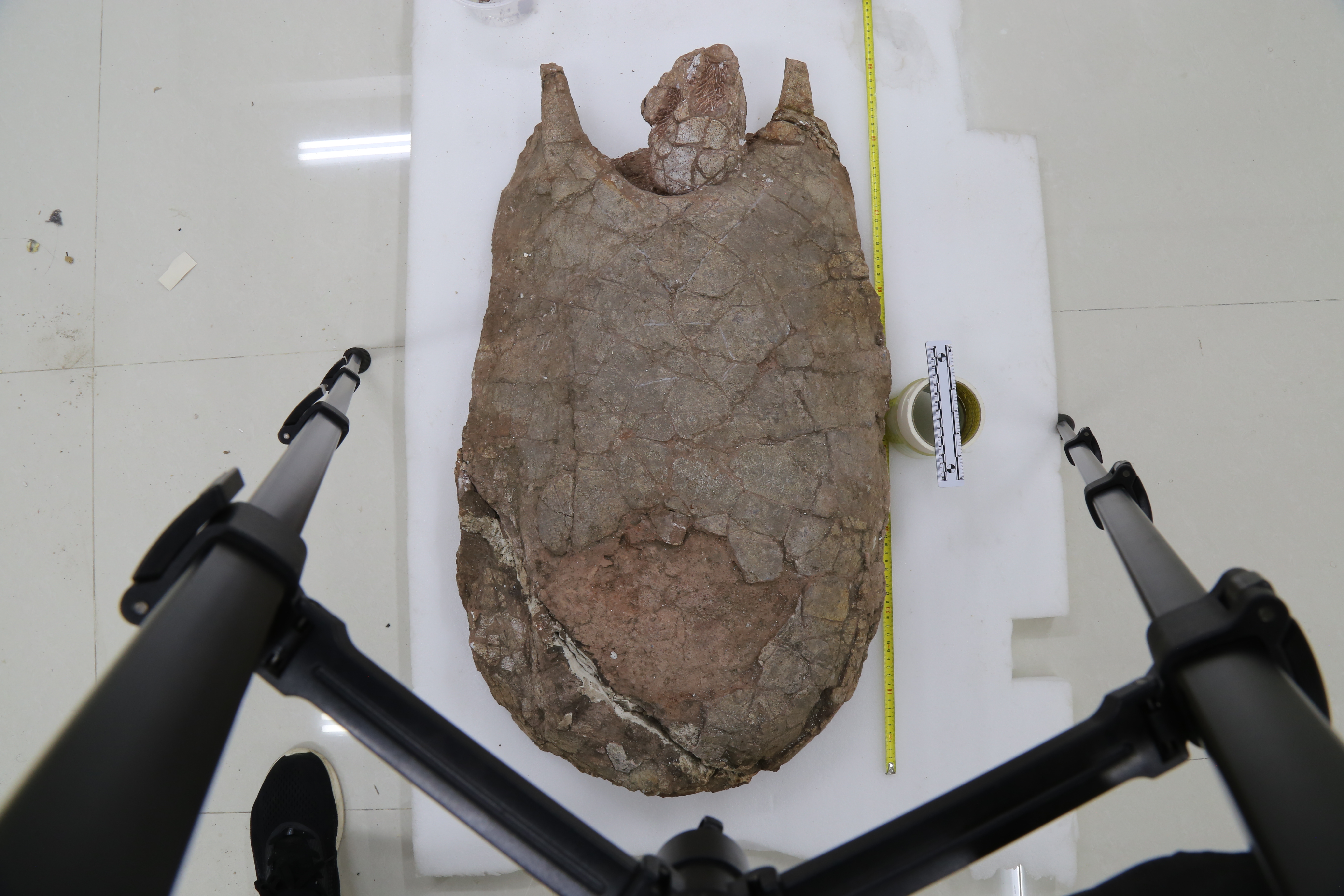

Supplement: Supplementary file 2 — Supplementary material 2: Original photos of SNHM 1558 [file 13358_2025_385_MOESM2_ESM.zip › original photos of SNHM1558/IMG_9794.JPG]

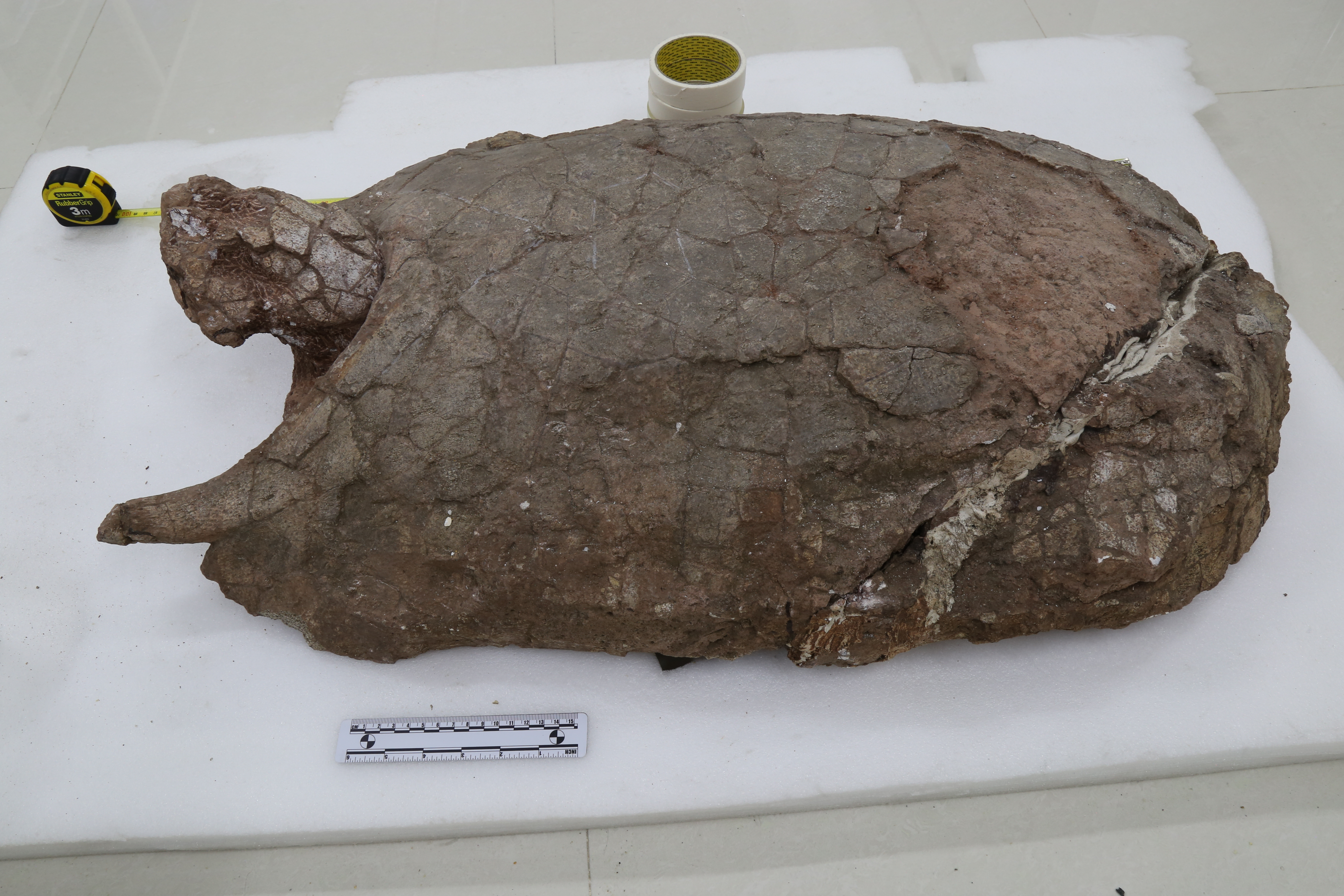

Supplement: Supplementary file 2 — Supplementary material 2: Original photos of SNHM 1558 [file 13358_2025_385_MOESM2_ESM.zip › original photos of SNHM1558/IMG_9800.JPG]

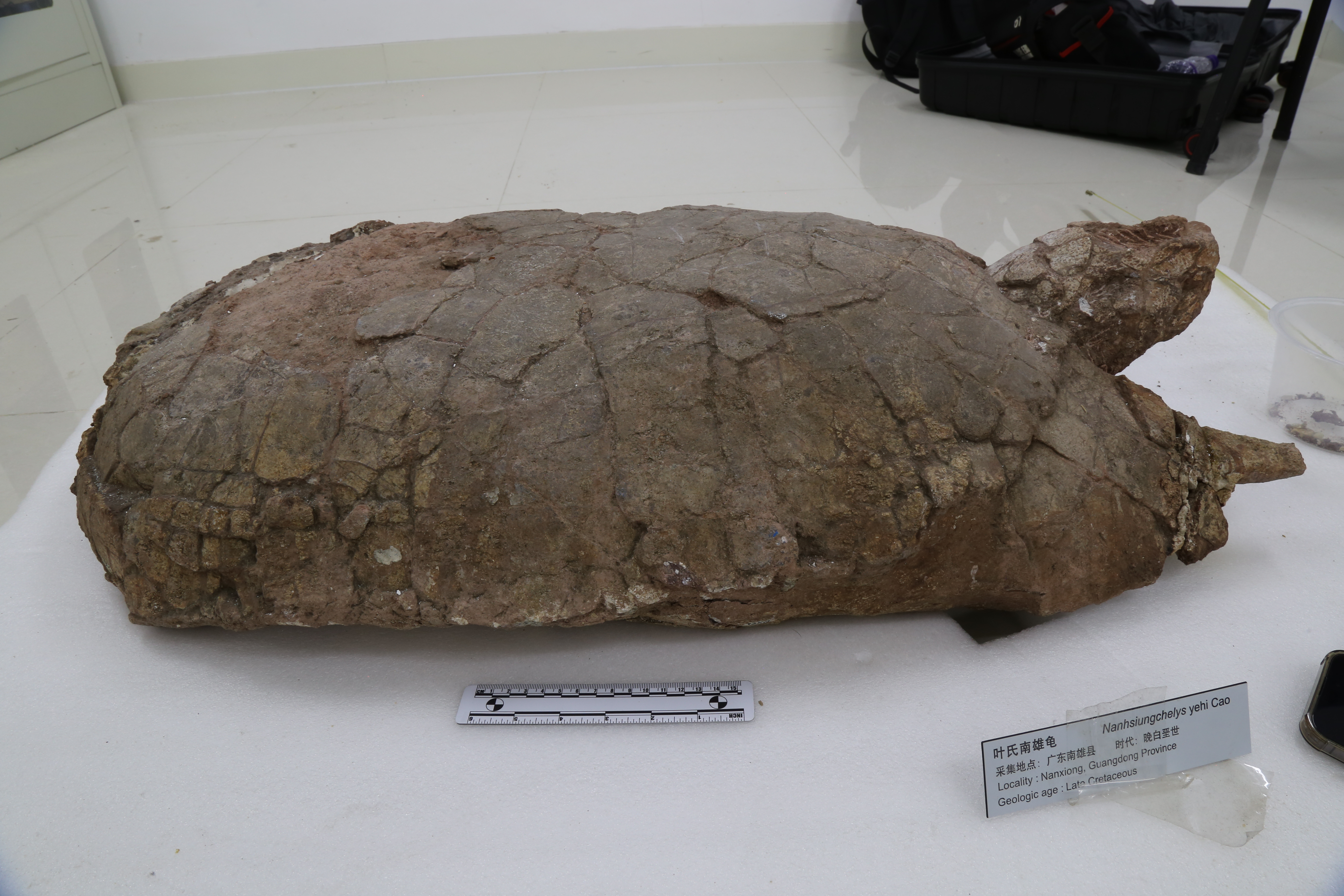

Supplement: Supplementary file 2 — Supplementary material 2: Original photos of SNHM 1558 [file 13358_2025_385_MOESM2_ESM.zip › original photos of SNHM1558/IMG_9832.JPG]

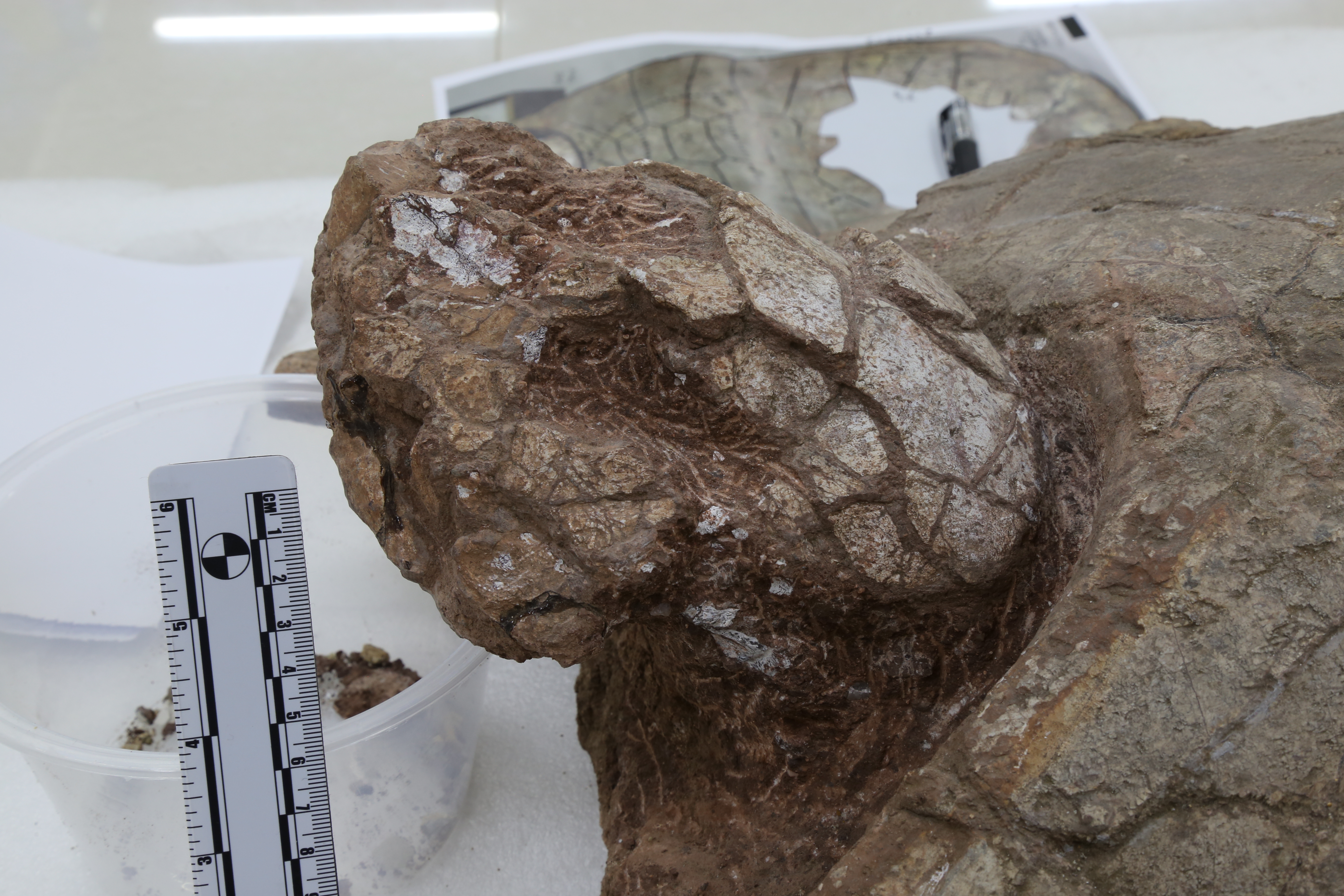

Supplement: Supplementary file 2 — Supplementary material 2: Original photos of SNHM 1558 [file 13358_2025_385_MOESM2_ESM.zip › original photos of SNHM1558/IMG_9847.JPG]

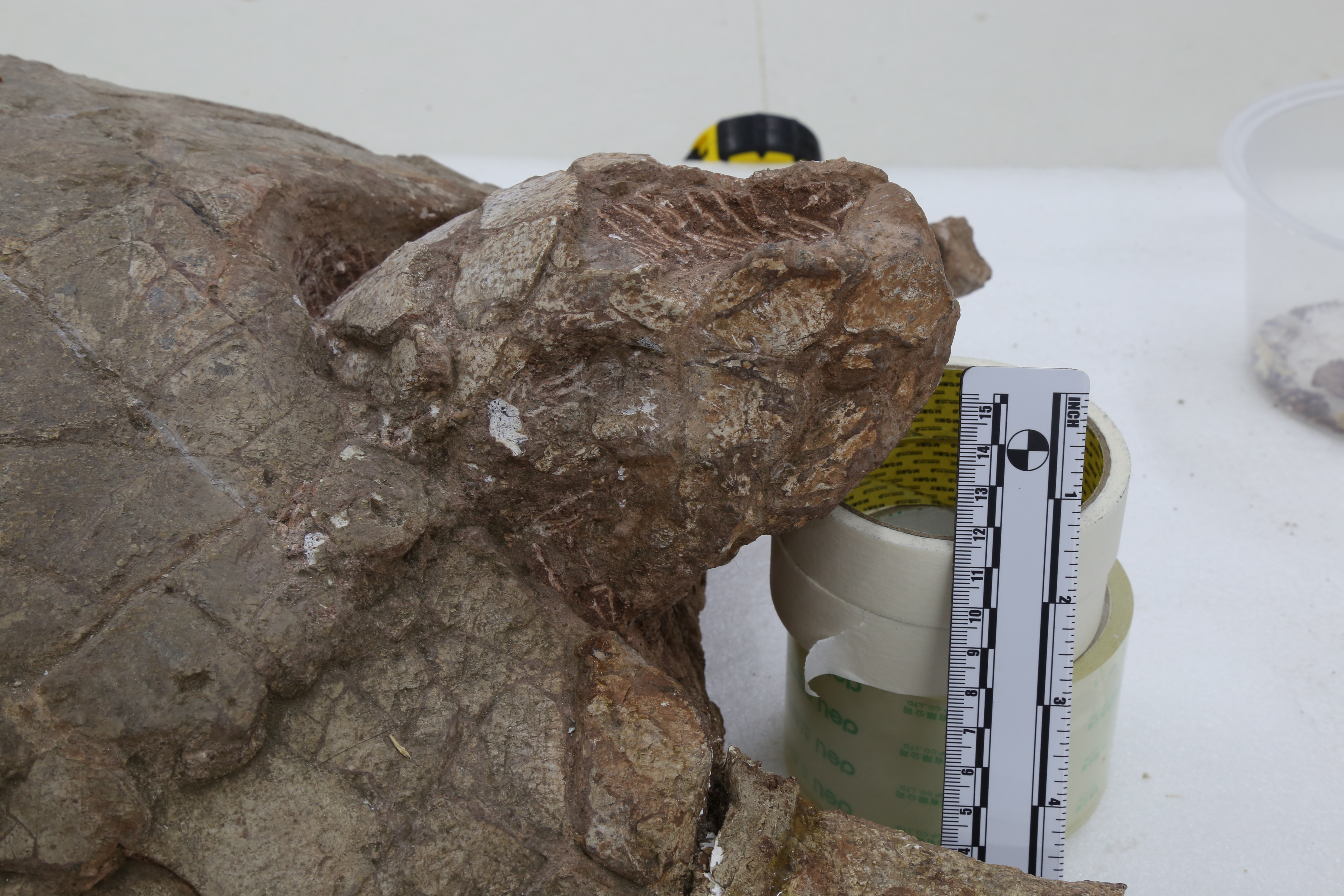

Supplement: Supplementary file 2 — Supplementary material 2: Original photos of SNHM 1558 [file 13358_2025_385_MOESM2_ESM.zip › original photos of SNHM1558/IMG_9855.JPG]

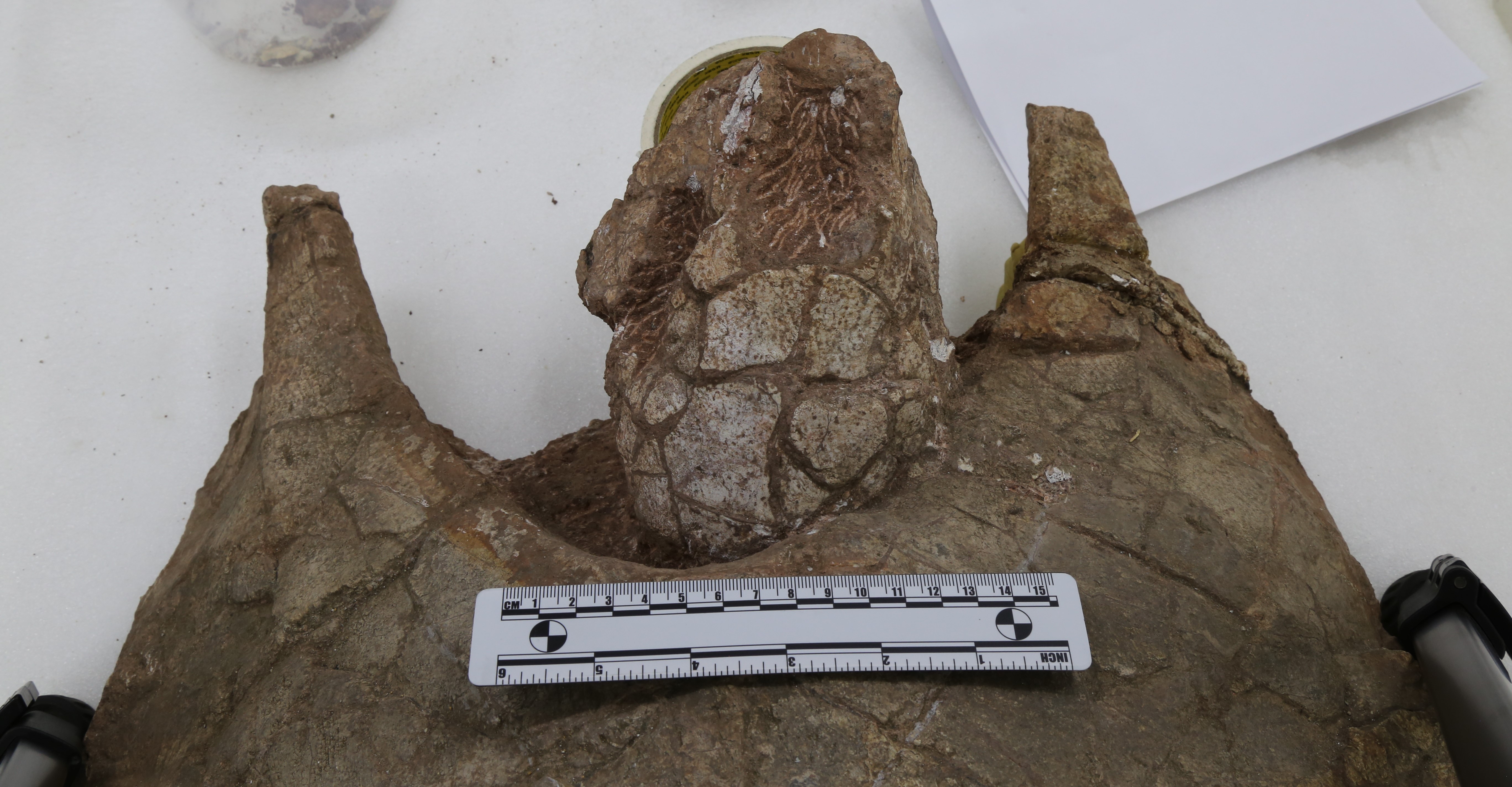

Supplement: Supplementary file 2 — Supplementary material 2: Original photos of SNHM 1558 [file 13358_2025_385_MOESM2_ESM.zip › original photos of SNHM1558/IMG_9856.JPG]

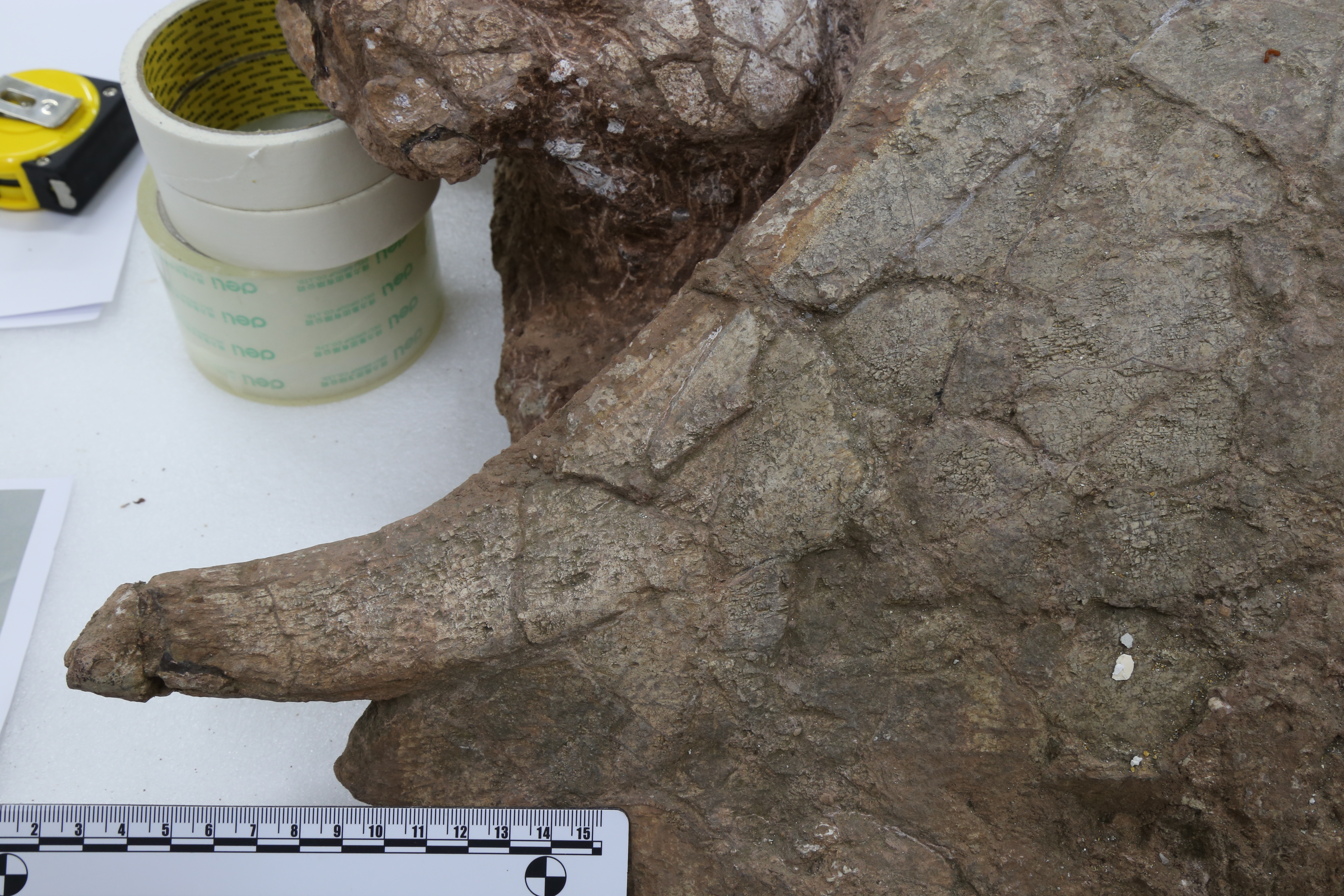

Supplement: Supplementary file 2 — Supplementary material 2: Original photos of SNHM 1558 [file 13358_2025_385_MOESM2_ESM.zip › original photos of SNHM1558/IMG_9864.JPG]

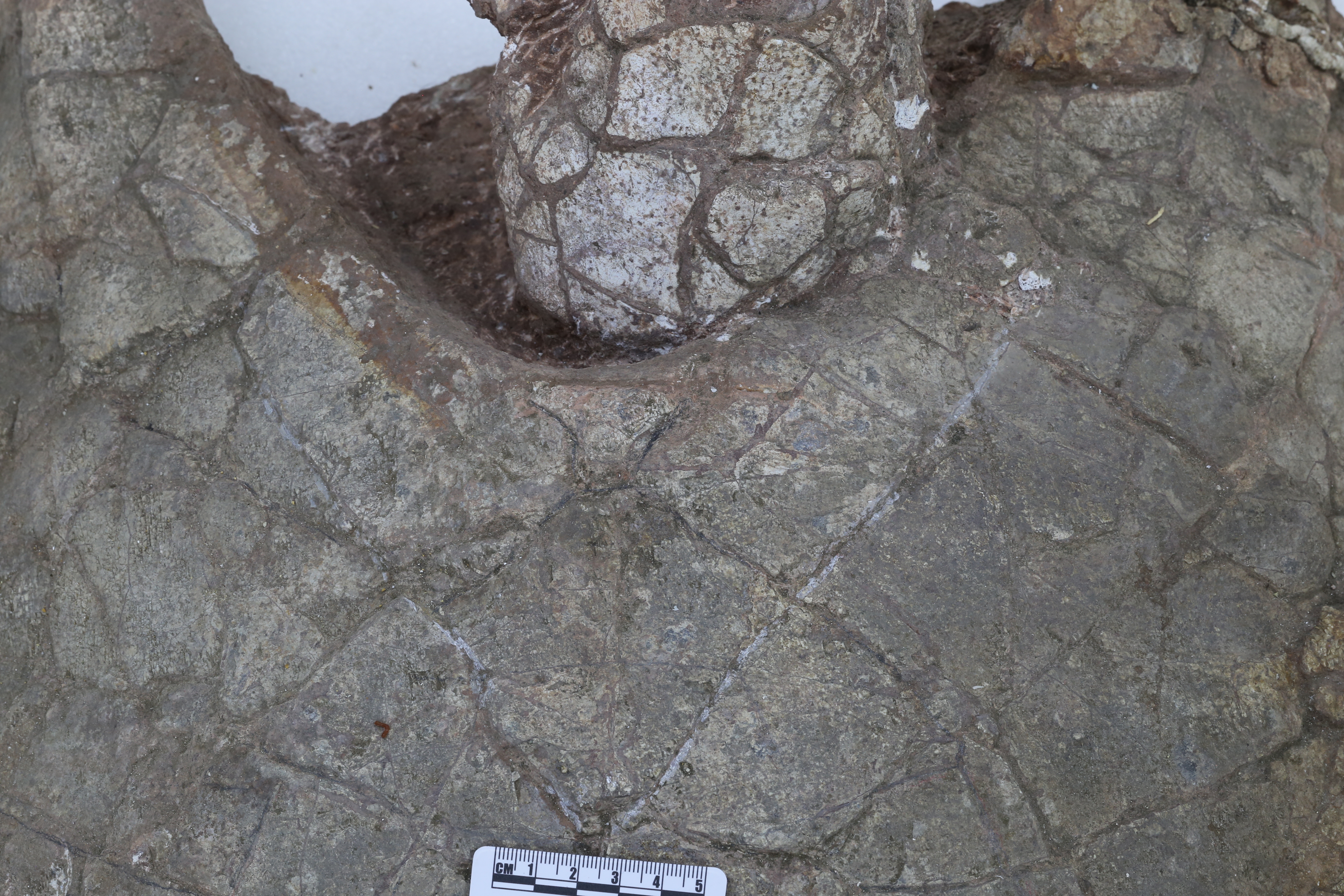

Supplement: Supplementary file 2 — Supplementary material 2: Original photos of SNHM 1558 [file 13358_2025_385_MOESM2_ESM.zip › original photos of SNHM1558/IMG_9886.JPG]

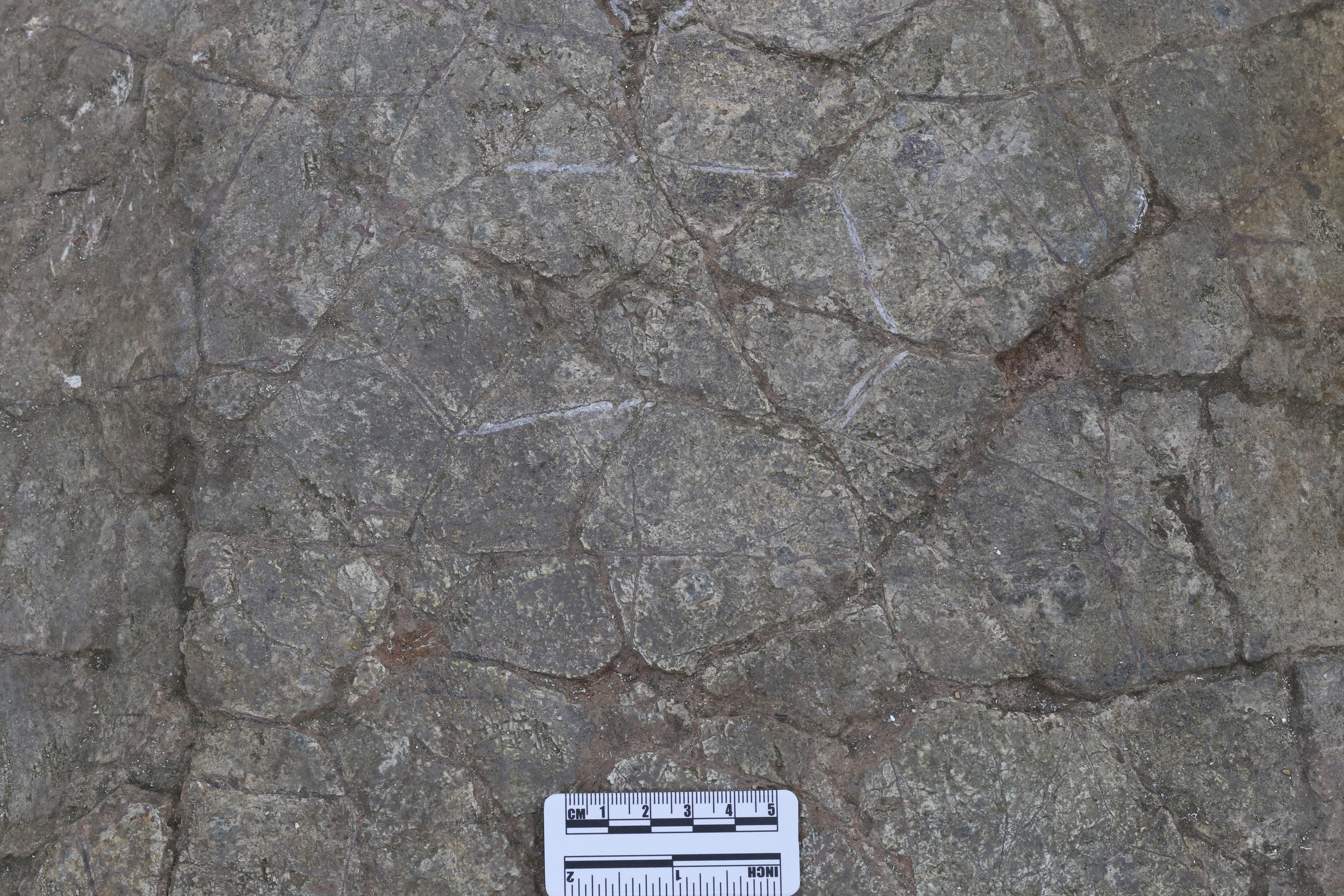

Supplement: Supplementary file 2 — Supplementary material 2: Original photos of SNHM 1558 [file 13358_2025_385_MOESM2_ESM.zip › original photos of SNHM1558/IMG_9887.JPG]

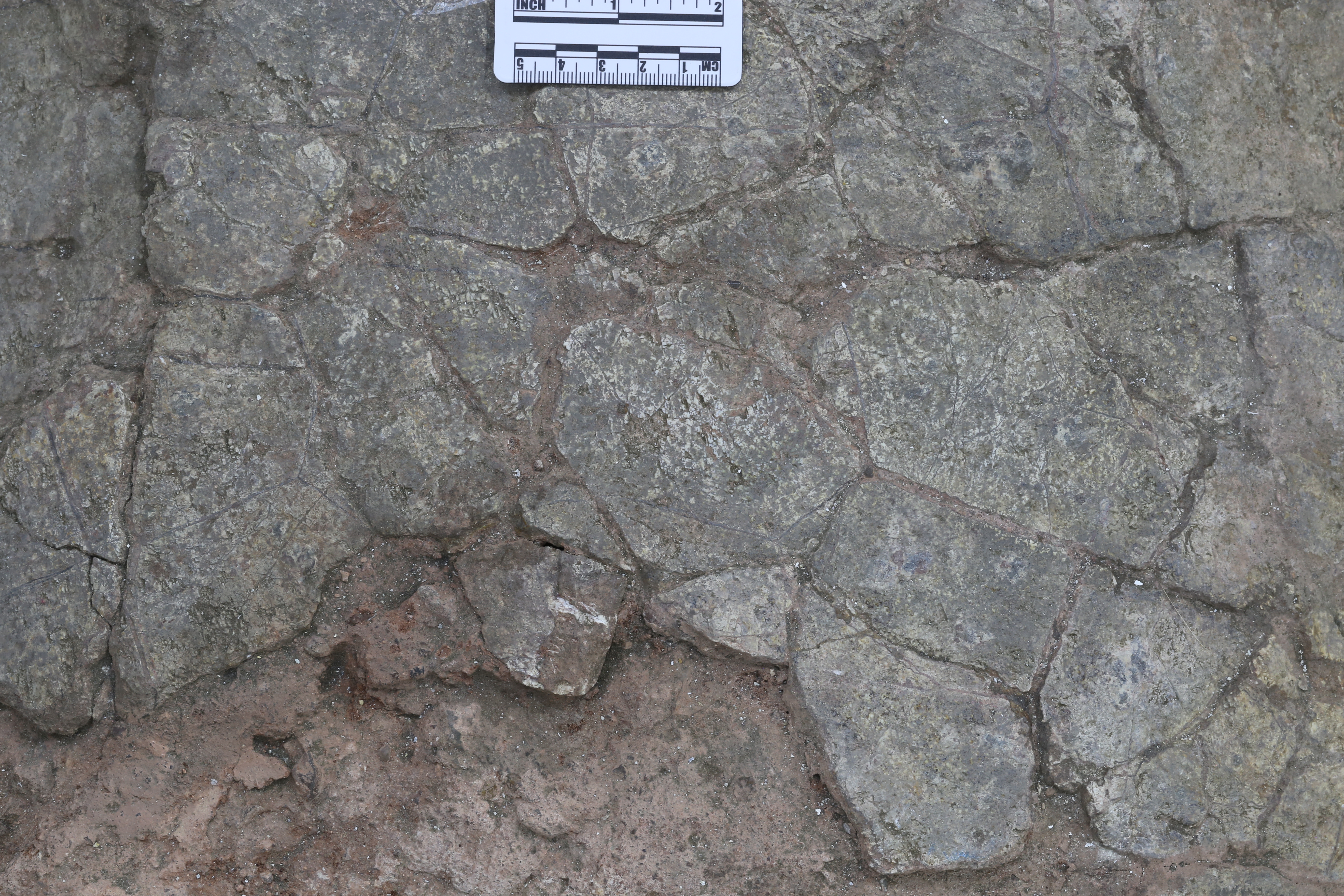

Supplement: Supplementary file 2 — Supplementary material 2: Original photos of SNHM 1558 [file 13358_2025_385_MOESM2_ESM.zip › original photos of SNHM1558/IMG_9888.JPG]

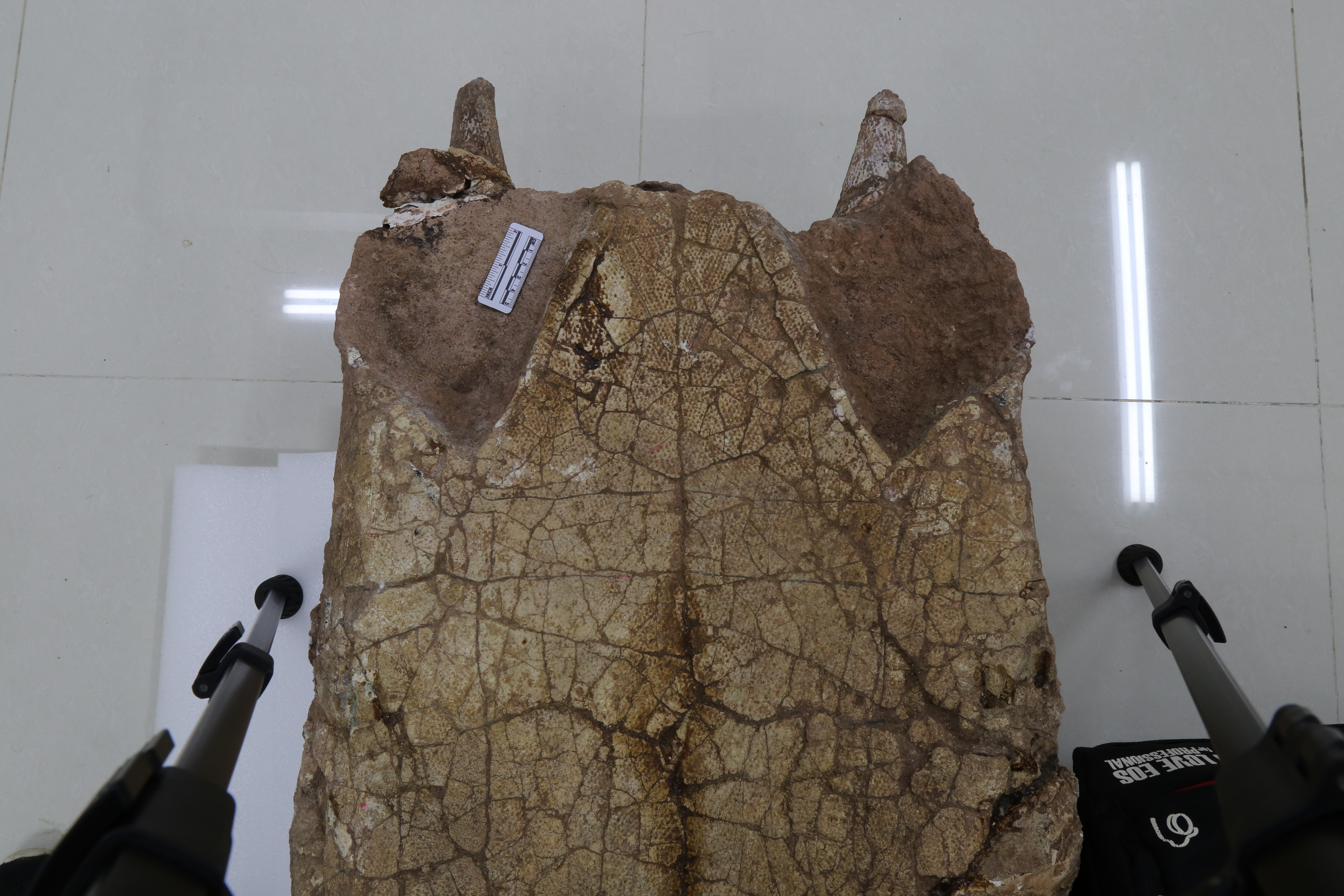

Supplement: Supplementary file 2 — Supplementary material 2: Original photos of SNHM 1558 [file 13358_2025_385_MOESM2_ESM.zip › original photos of SNHM1558/IMG_9898.JPG]

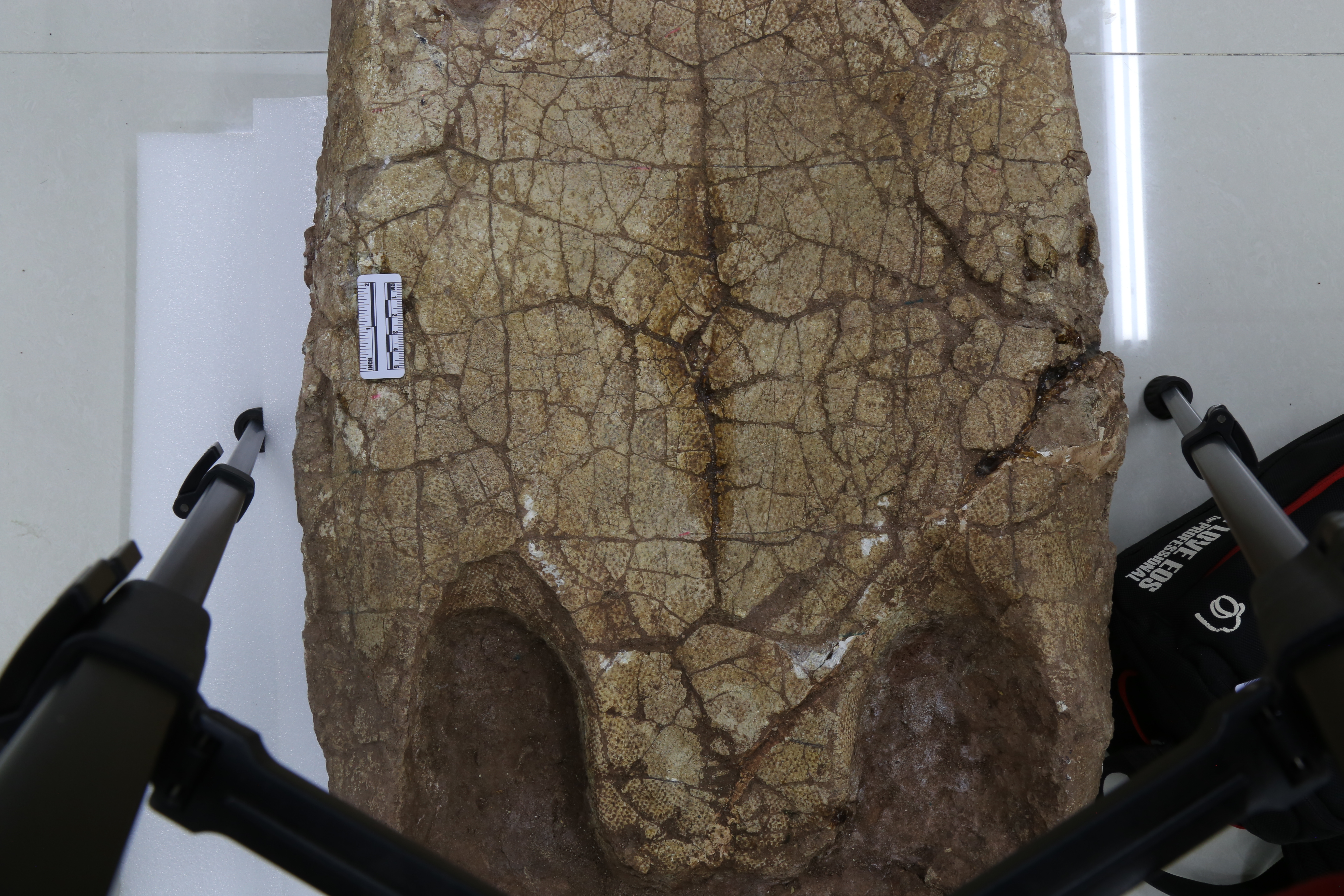

Supplement: Supplementary file 2 — Supplementary material 2: Original photos of SNHM 1558 [file 13358_2025_385_MOESM2_ESM.zip › original photos of SNHM1558/IMG_9902.JPG]

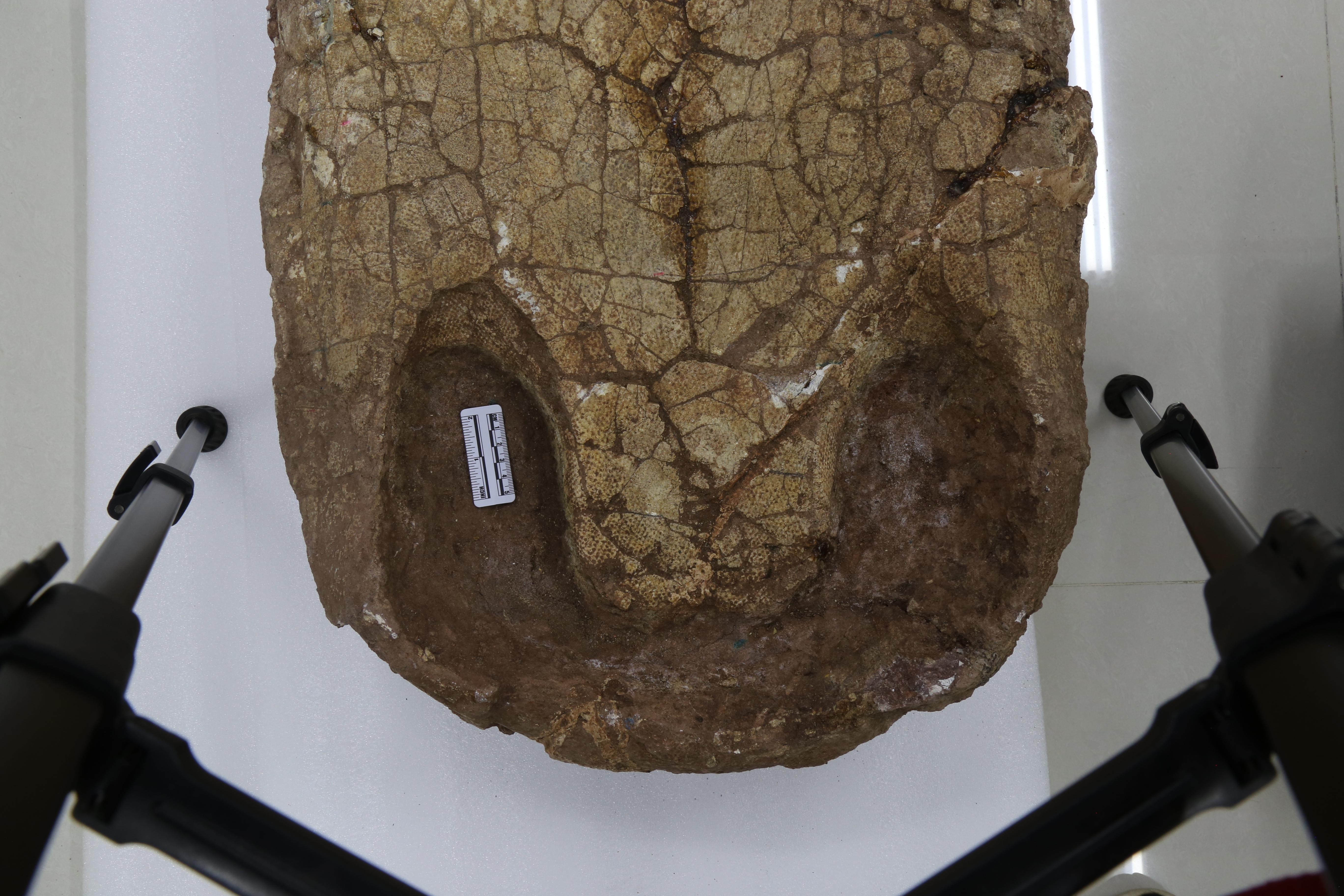

Supplement: Supplementary file 2 — Supplementary material 2: Original photos of SNHM 1558 [file 13358_2025_385_MOESM2_ESM.zip › original photos of SNHM1558/IMG_9904.JPG]

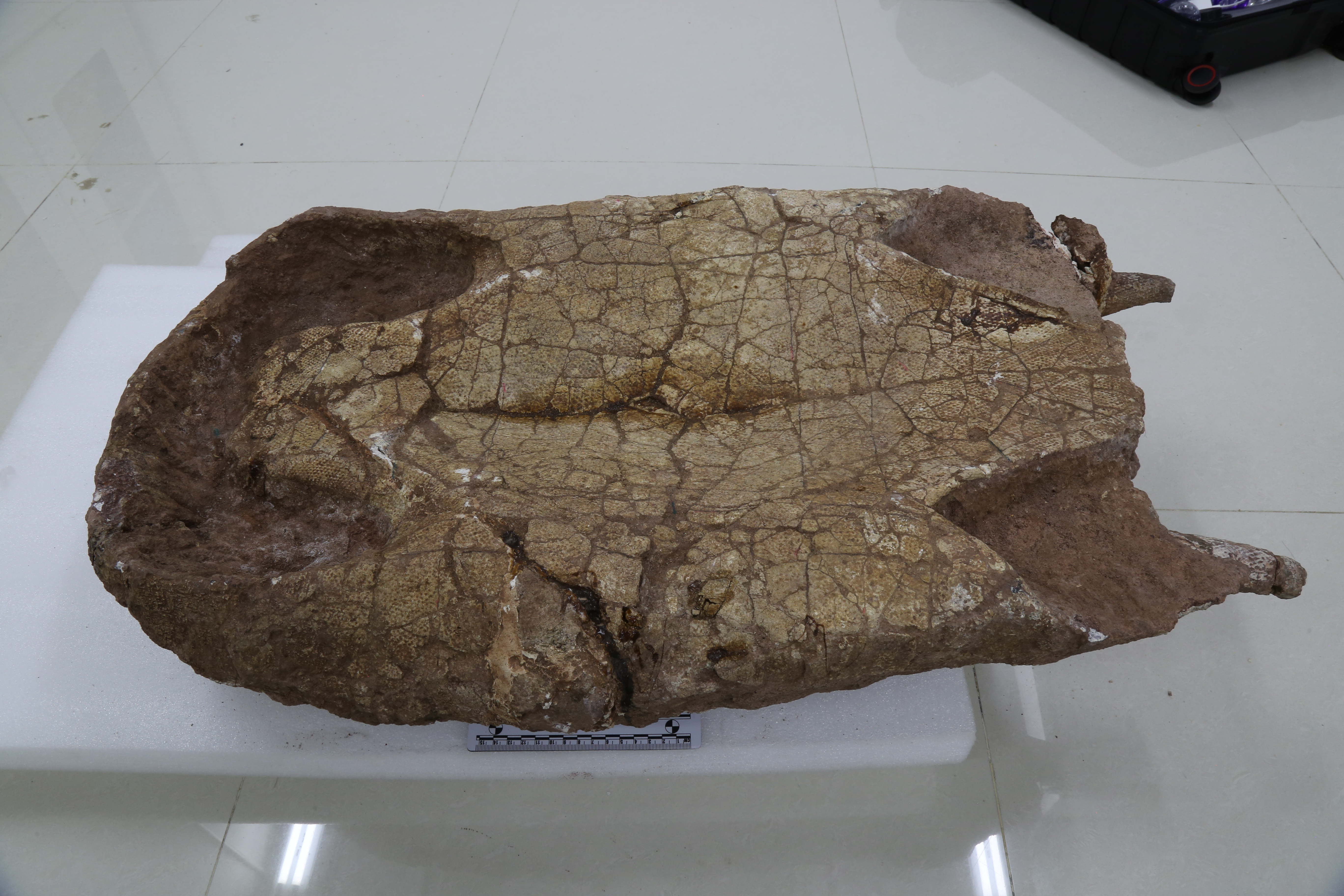

Supplement: Supplementary file 2 — Supplementary material 2: Original photos of SNHM 1558 [file 13358_2025_385_MOESM2_ESM.zip › original photos of SNHM1558/IMG_9917.JPG]

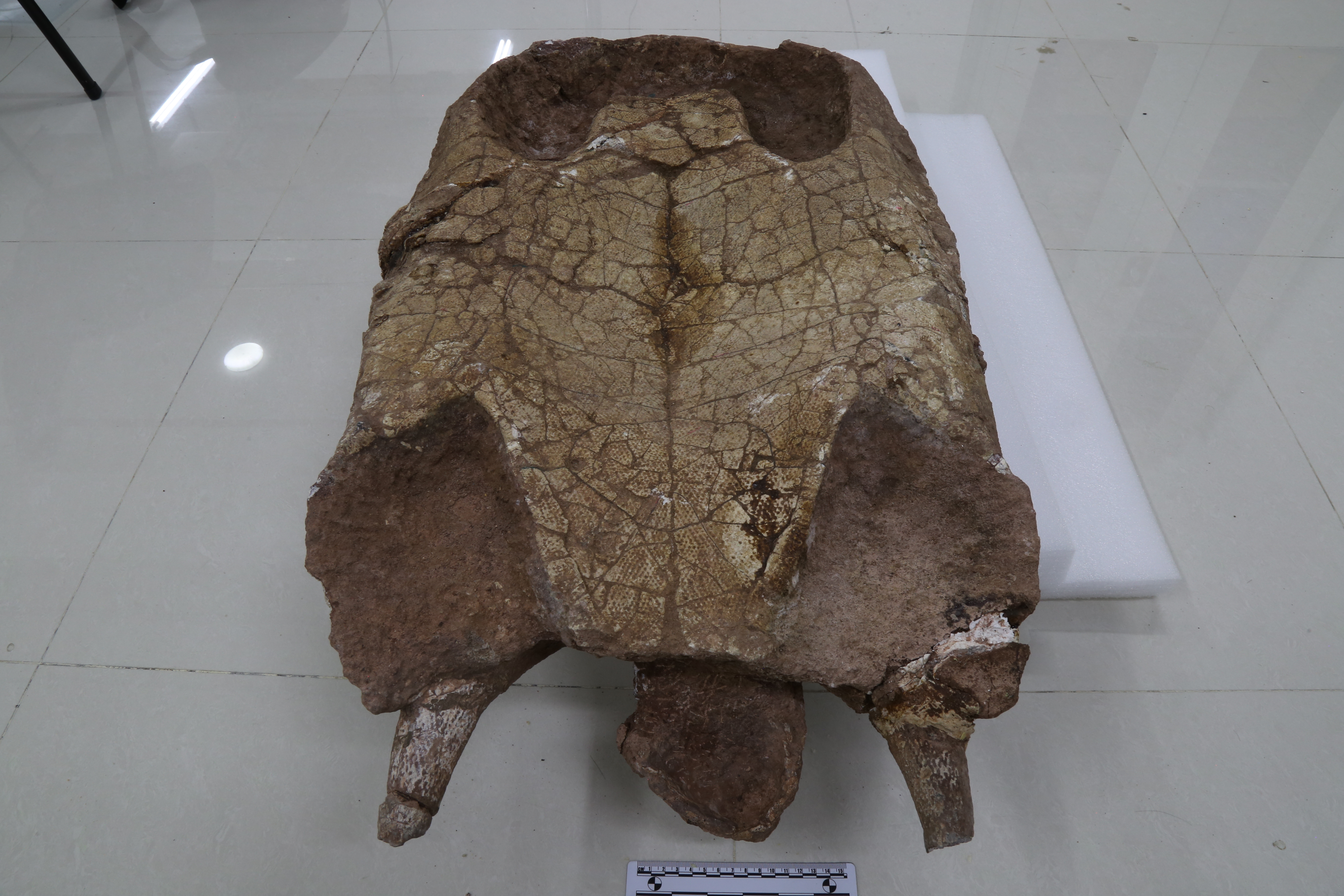

Supplement: Supplementary file 2 — Supplementary material 2: Original photos of SNHM 1558 [file 13358_2025_385_MOESM2_ESM.zip › original photos of SNHM1558/IMG_9924.JPG]
